# Identification and characterization of a set of monocot BAHD monolignol transferases

**DOI:** 10.1093/plphys/kiac035

**Published:** 2022-02-03

**Authors:** Rebecca A Smith, Emily T Beebe, Craig A Bingman, Kirk Vander Meulen, Alexis Eugene, Alexander J Steiner, Steven D Karlen, John Ralph, Brian G Fox

**Affiliations:** Great Lakes Bioenergy Research Center, University of Wisconsin–Madison, Madison, Wisconsin 53726, USA; Great Lakes Bioenergy Research Center, University of Wisconsin–Madison, Madison, Wisconsin 53726, USA; Department of Biochemistry, University of Wisconsin–Madison, Madison, Wisconsin 53706, USA; Department of Biochemistry, University of Wisconsin–Madison, Madison, Wisconsin 53706, USA; Great Lakes Bioenergy Research Center, University of Wisconsin–Madison, Madison, Wisconsin 53726, USA; Department of Biochemistry, University of Wisconsin–Madison, Madison, Wisconsin 53706, USA; Great Lakes Bioenergy Research Center, University of Wisconsin–Madison, Madison, Wisconsin 53726, USA; Great Lakes Bioenergy Research Center, University of Wisconsin–Madison, Madison, Wisconsin 53726, USA; Great Lakes Bioenergy Research Center, University of Wisconsin–Madison, Madison, Wisconsin 53726, USA; Great Lakes Bioenergy Research Center, University of Wisconsin–Madison, Madison, Wisconsin 53726, USA; Department of Biochemistry, University of Wisconsin–Madison, Madison, Wisconsin 53706, USA; Department of Biochemistry, University of Wisconsin–Madison, Madison, Wisconsin 53706, USA

## Abstract

Plant BAHD acyltransferases perform a wide range of enzymatic tasks in primary and secondary metabolism. Acyl-CoA monolignol transferases, which couple a CoA substrate to a monolignol creating an ester linkage, represent a more recent class of such acyltransferases. The resulting conjugates may be used for plant defense but are also deployed as important “monomers” for lignification, in which they are incorporated into the growing lignin polymer chain. *p*-Coumaroyl-CoA monolignol transferases (PMTs) increase the production of monolignol *p*-coumarates, and feruloyl-CoA monolignol transferases (FMTs) catalyze the production of monolignol ferulate conjugates. We identified putative FMT and PMT enzymes in sorghum (*Sorghum bicolor*) and switchgrass (*Panicum virgatum*) and have compared their activities to those of known monolignol transferases. The putative FMT enzymes produced both monolignol ferulate and monolignol *p*-coumarate conjugates, whereas the putative PMT enzymes produced monolignol *p*-coumarate conjugates. Enzyme activity measurements revealed that the putative FMT enzymes are not as efficient as the rice (*Oryza sativa*) control OsFMT enzyme under the conditions tested, but the SbPMT enzyme is as active as the control OsPMT enzyme. These putative FMTs and PMTs were transformed into Arabidopsis (*Arabidopsis thaliana*) to test their activities and abilities to biosynthesize monolignol conjugates for lignification in planta. The presence of ferulates and *p*-coumarates on the lignin of these transformants indicated that the putative FMTs and PMTs act as functional feruloyl-CoA and *p*-coumaroyl-CoA monolignol transferases within plants.

## Teaser

A group of identified BAHD acyltransferases function as feruloyl-CoA monolignol transferases and/or *p*-coumaroyl-CoA monolignol transferases in vitro and in planta.

## Introduction

Acyltransferases are a large family of enzymes with highly diverse functions in many organisms. In plants, enzymes that belong to the BAHD clades of the acyltransferase family play many key roles in primary and secondary metabolism. The BAHD family, so named for the first four enzymes that were discovered in this family, includes enzymes responsible for the synthesis of such products as fatty acids, lignin precursors, suberin, flavonoids, and many valuable natural products (for uses in, e.g. chemotherapy medications like Paclitaxel and Vinblastine, and compounds found in essential oils, nutraceuticals, and pigments) (St-Pierre and De Luca, 2000; D’Auria, 2006). As more genes are discovered, and the functions of the encoded enzymes are identified and verified in planta, the list of substrates and activities for this diverse group of enzymes grows in both importance and utility (Bontpart et al., 2015; Eudes et al., 2016a). 

**Figure 1:**
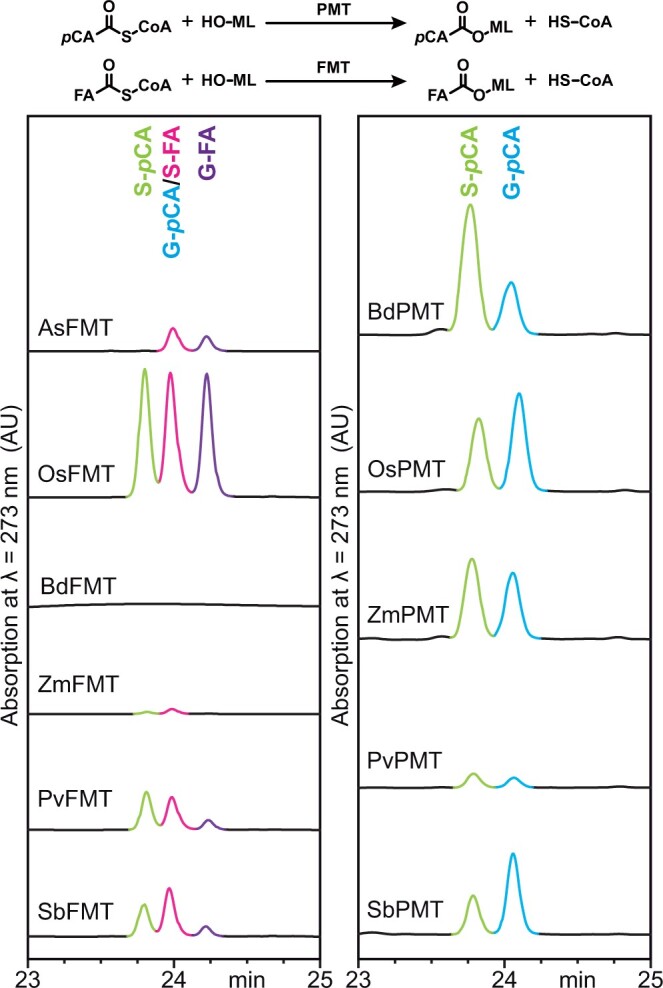
Results of feruloyl-CoA monolignol transferase (FMT) and *p*-coumaroyl-CoA monolignol transferase (PMT) competition enzyme assays. The general mechanism of FMT and PMT enzyme activity is shown at the top of the figure. Each FMT or PMT was tested for activity with feruloyl-CoA or *p*-coumaroyl-CoA and the three monolignols (ML) pooled together as the alcohol acceptors. Sinapyl ferulate (S-FA) peaks are outlined in pink, coniferyl ferulate (G-FA) peaks are outlined in purple, sinapyl *p*-coumarate (S-*p*CA) peaks are outlined in light green, and coniferyl *p*-coumarate (G-*p*CA) peaks are outlined in light blue.

One of the more recently described class of BAHD enzymes is the acyl-CoA monolignol transferases (Withers et al., 2012; Marita et al., 2014; Wilkerson et al., 2014). These enzymes couple acyl-CoA donors, such as *p*-coumaroyl-CoA or feruloyl-CoA, with hydroxycinnamyl alcohols (lignin monomer, monolignols) as acceptors, yielding monolignol ester conjugates (Figure 1). The canonical monolignols are: *p*-coumaryl alcohol (giving rise to H-units in lignin), coniferyl alcohol (G-units), and sinapyl alcohol (S-units). The product monolignol conjugates represent an alternate chemical flux through which monolignol precursors can be channeled. Monolignol conjugates often function as lignin “monomers” and participate in lignification; they are hypothesized to be exported into the cell wall by the same simple diffusion mechanism as monolignols (Vermaas et al., 2019). Typically, the acyl group remains pendent and can be liberated from the lignin polymer backbone by hydrolysis of the ester linkage. Under some conditions, both halves of the monolignol conjugate can participate in lignification, with the ester bond fully incorporated into the lignin polymer and generating so-called “zip-lignins”, either through a linear linkage (extending the polymer chain) or by crosslinking two lignin polymer chains (Grabber et al., 2002; Ralph, 2010; Rencoret et al., 2013; Wilkerson et al., 2014; Lu et al., 2015; Smith et al., 2015; Kaal et al., 2018). As an alternative to lignin polymerization, the monolignol conjugates, like many other phenolic compounds, can be glycosylated and stored in cell vacuoles (Dima et al., 2015). An investigation of a broad range of plant species across the Spermatophytes (gymnosperms and angiosperms) revealed that monolignol conjugates are naturally produced by many species (Hatfield et al., 2009; Ralph, 2010; Karlen et al., 2016, 2018). Gymnosperms do not appear to produce monolignol conjugates; however a few select eudicots, such as poplar (*Populus* spp.) and Chinese Angelica (*Angelica sinensis*), produce small amounts of the monolignol ferulates (ML-FA), as products of monolignols coupling to feruloyl-CoA, an intermediate in the lignin biosynthetic pathway (Karlen et al., 2016). Members of the Poaceae family (e.g. Brachypodium [*Brachypodium distachyon*], maize [*Zea mays*], rice [*Oryza sativa*], switchgrass [*Panicum virgatum*], and sorghum [*Sorghum bicolor*]), in contrast, all produce monolignol ferulates and large quantities of monolignol *p*-coumarates (ML-*p*CA), which are the product of coupling monolignols with *p*-coumaroyl-CoA, another intermediate in the lignin biosynthetic pathway (Ralph et al., 1994; Withers et al., 2012; Marita et al., 2014; Petrik et al., 2014; Karlen et al., 2016, 2018). The BAHD acyltransferases responsible for the synthesis of monolignol ferulates are referred to as feruloyl-CoA monolignol transferases (FMTs), and those that synthesize monolignol *p*-coumarates are *p*-coumaroyl-CoA monolignol transferases (PMTs). Understanding FMT and PMT activity in plants is of great interest because of their prevalence in agriculturally important crops, in model organisms, and/or in potential biorefinery feedstocks (Withers et al., 2012; Karlen et al., 2016).

The first FMT enzyme identified was native to *Angelica sinensis* (AsFMT) and, when transformed into poplar (*Populus alba* × *grandidentata*), was able to confer upon the plant the ability to produce larger quantities of monolignol ferulates (Wilkerson et al., 2014). Because of the nature of ML-FAs, which have the ability to integrate into the lignin polymer and introduce chemically labile bonds into the polymer, the digestibility of the poplar wood under mild pretreatment or harsh pulping was significantly improved (Kim et al., 2017; Zhou et al., 2017; Bhalla et al., 2018). Similar results were observed when *AsFMT* was transformed into the model plant Arabidopsis (*Arabidopsis thaliana*) (Smith et al., 2017). Subsequently, Bartley’s group identified a highly active FMT enzyme that produces ML-FAs in rice (OsFMT/AT5) (Karlen et al., 2016). Because of the prevalence of ML-*p*CA in grass lignins, and the relative ease of detecting the incorporation of monolignol *p*-coumarates into lignin by thioacidolysis (Grabber et al., 1996) or the derivatization followed by reductive cleavage (DFRC) method (Lu and Ralph, 1999; Petrik et al., 2014; Regner et al., 2018), many more PMT enzymes responsible for the native production of ML-*p*CA on monocot lignins have been identified and characterized. These include, ZmPMT/*p*CAT (maize), OsPMT (rice), and BdPMT (Brachypodium) (Withers et al., 2012; Marita et al., 2014; Petrik et al., 2014; Smith et al., 2015; Sibout et al., 2016; Karlen et al., 2020).

There is a strong desire in the lignin biofuel and bioproduct field to manipulate the native levels of monolignol conjugates produced in potential biofuel crops such as sorghum and switchgrass. There is also a movement away from genetically modifying plants to ensure that optimized biofuel crops can be grown in the field anywhere around the world, which makes it imperative to discover all the native FMT and PMT enzymes for these species of interest. The focus of this study was to identify previously unknown FMT and PMT enzymes from *Z.* *mays*, *S.* *bicolor*, and *P.* *virgatum* and to characterize the activities of those enzymes in vitro and in planta, using *A.* *thaliana* as a model system. The identification of additional monolignol transferases also allowed us to ascertain which FMT and PMT enzymes identified to date are the most specific for a given activity, and thus enable genome-guided plant breeding.

## Results

### Identification of enzymes of interest

To identify additional grass monolignol transferase enzymes, the assumption was made that the acyltransferases with activities of interest would have high amino acid sequence similarity to previously identified monocot FMTs and PMTs. The rice FMT (OsFMT) and PMT (OsPMT) are known and these BAHD enzymes served as the reference sequences for the identification of putative FMT and PMT enzymes from *S.* *bicolor* (sorghum), *Z.* *mays* (maize), and *P.* *virgatum* (switchgrass). The putative FMTs identified from the sorghum, switchgrass, and maize genomes retained the BAHD acyltransferase conserved residues and had >70% amino acid identity compared to the OsFMT enzyme (Table 1). A putative *B.* *distachyon* FMT (BdFMT) enzyme was also identified based on 70% sequence identity to OsFMT. Similarly, the putative PMT enzymes had ∼60% amino acid identity to the OsPMT enzyme (Table 1). Interestingly the putative FMT enzymes only had ∼25% amino acid identity compared to the AsFMT enzyme. This is similar to the 23% amino acid identity between OsFMT and AsFMT. This disparity in amino acid sequence similarity between grass monocot and eudicot (AsFMT) acyltransferases suggests that only minimal primary sequence identity, including the conserved HXXXD motif, is needed to retain enzymatic function while potentially allowing broadened substrate specificity.

**Table 1:** Sequence similarity/identity between control FMT and PMT enzymes and the identified monolignol transferases

				% Identity		
Enzyme	Species	GenBank/Phytozome ID	AA length	versus *As*FMT	versus *Os*FMT	versus *Os*PMT
AsFMT	*Angelica sinensis*	XM_017385232.1	442	100% (442/442)	23% (95/422)	22% (77/354)
OsFMT (OsAT5)	*O. sativa*	XM_015785190.2	433	25% (49/199)	100% (433/433)	57% (250/439)
ZmFMT	*Zea mays*	Zm00001d035246_T001	434	27% (34/128)	73% (318/436)	57% (236/412)
PvFMT	*P. virgatum*	Pavir.3KG495300	436	21% (74/351)	76% (331/437)	56% (250/444)
SbFMT	*S. bicolor*	XM_002441921.2	441	26% (52/203)	77% (335/436)	57% (255/444)
BdFMT	*Brachypodium distachyon*	XP_003575887.1	443	22% (91/421)	69% (304/443)	56% (249/446)
OsPMT	*O. sativa*	XM_015765814.2	440	29% (36/123)	57% (248/438)	100% (440/440)
PvPMT	*P. virgatum*	XM_039943249.1	428	24% (98/408)	53% (230/433)	62% (274/439)
SbPMT	*S. bicolor*	XM_002439193.2	437	23% (93/403)	54% (232/432)	64% (282/442)

### In vitro characterization of the putative monocot monolignol acyltransferases

Genes encoding putative acyltransferase enzymes were synthesized into an SP6 promoter-containing plasmid by GenScript Corporation and were expressed using wheatgerm cell-free translation (Supplemental Figure S1 shows denaturing gel analysis). A cell-free translation reaction with no mRNA template was assayed as a negative control to identify potential background transferase activity in the wheatgerm extract, and none was observed. As positive controls, the AsFMT, OsFMT, OsPMT, BdPMT, and ZmPMT enzymes were synthesized and 10 µL aliquots of the translation reaction were used in screening assays (Table 2).

**Table 2:** Reactivity of each acyltransferase enzyme with CoA donors and monolignol alcohol acceptors

	Reactivity with monolignols (H–OH, G–OH, and S–OH)						
Enzyme	Ac-CoA	B-CoA	*p*HBA-CoA	*p*CA-CoA	Caff-CoA	FA-CoA	References
AsFMT	**–**	**–**				**+++**	Wilkerson et al. (2014)
OsFMT (OsAT5)				**+**		**+++**	Karlen et al. (2016)
ZmFMT				**–**		**+**	
PvFMT				**–**		**+++**	
SbFMT				**–**		**+++**	
BdFMT				**–**		**–**	
OsPMT	***+***			**++**	**+**	**+**	Withers et al. (2012)
PvPMT				**+**		**–**	
SbPMT				**+**		**–**	

Ac-CoA, acetyl-CoA; B-CoA, benzoyl-CoA; *p*HBA-CoA, *p*-hydroxybenzoyl-CoA; *p*CA-CoA, *p*-coumaroyl-CoA; Caff-CoA, caffeoyl-CoA; and FA-CoA, feruloyl-CoA.

AsFMT was assayed for activity with feruloyl-CoA and/or *p*-coumaroyl-CoA with the three monolignols in a mixture (*p*-coumaryl alcohol, H–OH; coniferyl alcohol, G–OH; and sinapyl alcohol, S–OH). The reaction was allowed to proceed for 30 min, following which the reaction was stopped and products were detected by LCMS. The assays resulted in the production of coniferyl ferulate (G-FA) and sinapyl ferulate (S-FA) (Figure 1). OsFMT was subjected to similar assays, resulting in the production of G-FA, S-FA, and sinapyl *p*-coumarate (S-*p*CA). This indicates that the OsFMT enzyme has a more relaxed substrate specificity than the AsFMT enzyme (Figure 1 and Table 2).

OsPMT, ZmPMT, and BdPMT were used as positive controls for PMT activity. Each enzyme was tested for activity with the monolignols and feruloyl-CoA and/or *p*-coumaroyl-CoA. All of the control PMT enzymes preferentially produced monolignol *p*-coumarates, specifically coniferyl *p*-coumarate (G-*p*CA) and S-*p*CA (Figure 1 and Table 2). The inability to produce monolignol ferulates, either in substrate competition assays or in individual enzyme assays, indicates that the PMT control enzymes have stricter substrate specificity than the FMT control enzymes.

The putative FMT enzymes (SbFMT, PvFMT, and ZmFMT) showed similar substrate preferences to the OsFMT enzyme. When tested in individual enzyme assays with either feruloyl-CoA or *p*-coumaroyl-CoA as the CoA donor and the three monolignols as alcohol acceptors (i.e. one CoA thioester donor and all monolignol alcohol acceptors), the enzymes were capable of coupling one or more of the three monolignols to feruloyl-CoA to produce monolignol ferulates (Supplemental Figure S2). The FMT enzymes were also able to couple coniferyl alcohol and sinapyl alcohol to *p*-coumaroyl-CoA to produced G-*p*CA or S-*p*CA (Supplemental Figure S3). In the competition assays (assays with both CoA thioester donors and all three monolignol alcohol acceptors), the FMT enzymes preferentially produced G-FA, S-FA, and S-*p*CA (Figure 1 and Table 2). These enzymes therefore seemed to have the same substrate promiscuity as the control OsFMT enzyme. The putative BdFMT enzyme was also tested for activity as a feruloyl-CoA and/or *p*-coumaroyl-CoA monolignol transferase. Despite the high sequence similarity between BdFMT and OsFMT, the BdFMT did not have any detectable activity as an FMT under the conditions tested in this study (i.e. did not produce detectable levels of monolignol ferulate or *p*-coumarate products in 30 min) (Figure 1 and Table 2). This observation highlights that sequence similarity alone may not be sufficient to predict whether an individual enzyme is active or has a unique substrate specificity, a finding that is supported by other studies involving closely related acyltransferases (Beekwilder et al., 2004). The similarity between the putative grass FMT enzymes (SbFMT, PvFMT, and ZmFMT) and OsFMT, and the differences between the grass FMT enzymes and AsFMT, a representative eudicot FMT, suggests the trend that eudicot FMTs have greater substrate specificity than monocot FMTs. As the crystal structures of these enzymes are not available, it is unclear how differences between eudicot and monocot enzymes relate to their substrate preferences and promiscuity.

The putative PMT enzymes (SbPMT and PvPMT) were also tested for activity with *p*-coumaroyl-CoA, feruloyl-CoA, and the monolignols. As with the control PMT enzymes, the putative PMT enzymes preferentially used *p*-coumaroyl-CoA as an acyl donor and produced G-*p*CA and S-*p*CA (Figure 1 and Table 2). In both the individual enzyme assays and the competition assays, the PMT enzymes did not use feruloyl-CoA as an acyl donor (Figure 1, Table 2, and Supplemental Figure S3). The grass PMT enzymes therefore exhibit greater substrate specificity than the grass FMT enzymes. The rice OsFMT and OsPMT enzymes share about 50% primary sequence identity and it is hypothesized that the differences in the active sites between the FMT and PMT enzymes relate to the substrate specificity or promiscuity.

Enzyme activity measurements, normalized to an internal standard and the amount of enzyme added to the assay, were performed to determine the relative efficiency of producing various monolignol conjugates. Concentrations of soluble enzyme produced in the translation reaction were quantified using denaturing electrophoresis and an established BioRad stain-free analysis method (Makino et al., 2014; Supplemental Figure S4). Based on this quantitation of the translation products, a similar amount of acyltransferase was added to each reaction so that the mass spectral ion counts observed for each product at the endpoint time of the assay could be normalized to the amount of enzyme present. This provides the basis for our comparisons of the activities between different enzymes (Table 3).

**Table 3:** Enzyme activity of the control and FMT and PMT enzymes

		Normalized ML-FA or ML-*p*CA product area/µg enzyme							
CoA donor	Alcohol acceptor	SbFMT	PvFMT	ZmFMT	AsFMT	OsFMT	SbPMT	PvPMT	OsPMT
FA-CoA	S–OH	3.19	0.95	0.8	0.7	5.11	Trace	Trace	0.63
	G–OH	9.38	3.71	1.76	1.64	15.2	Trace	Trace	Trace
	H–OH	3.23	3.08	0.57	Trace	6.59	Trace	ND	Trace
*p*CA-CoA	S–OH	2.06	2.99	Trace	ND	13.74	2.06	trace	2.35
	G–OH	Trace	Trace	ND	ND	1.46	6.7	1.55	1.73
	H–OH	ND	ND	ND	ND	ND	7.5	2.17	8.22

Reactions contained 1 mM CoA thioester, 1 mM monolignol alcohol, and equivalent amounts of acyltransferase as determined by stain-free gel analysis (see Supplemental Figure S4). Product areas were determined by measuring the area under the monolignol ferulate (ML-FA) or monolignol *p*-coumarate (ML-pCA) peak at the maximum absorption wavelength for the products (324 nm and 309 nm, respectively). FA-CoA, feruloyl-CoA; *p*CA-CoA, *p*-coumaroyl-CoA; H–OH, *p*-coumaryl alcohol; G–OH, coniferyl alcohol; S–OH, sinapyl alcohol; ND, no product detected; trace, product area is at or below the limit of detection (<5 mAu).

Each enzyme was tested with each CoA donor and monolignol independently, resulting in six different reactions (feruloyl-CoA and each monolignol, *p*-coumaroyl-CoA and each monolignol). Of the FMT enzymes, OsFMT had the strongest activity with the three monolignols and feruloyl-CoA (Table 3 and Supplemental Figure S5). Under the conditions used in this study, amongst the putative FMT enzymes, SbFMT had the highest activity, followed by PvFMT and ZmFMT, but the activities of all these enzymes were lower than that of OsFMT (Table 3 and Supplemental Figure S5). ZmFMT and AsFMT were the least active enzymes and produced only S-FA under these assay conditions. Although OsFMT is the most active FMT enzyme, it also had the highest production of S-*p*CA out of any enzyme, further demonstrating the relaxed substrate preferences of OsFMT (Supplemental Figure S5). As has previously been demonstrated, the OsPMT enzyme had highest activity with *p*-coumaryl alcohol and *p*-coumaroyl-CoA (Withers et al., 2012) over the other PMT enzymes. In this study, SbPMT also had strong activity with *p*-coumaryl alcohol and *p*-coumaroyl-CoA but was by far the most reactive with the coniferyl alcohol and *p*-coumaroyl-CoA substrates (Table 3 and Supplemental Figure S5). The activity of the PMT enzymes with feruloyl-CoA was low, and the only activity above the limit of detection was that of OsPMT with sinapyl alcohol.

### Characterization of the putative monocot monolignol acyltransferases in planta

To test the activity of putative FMT and PMT enzymes in planta to verify their activity as monolignol transferases, genes encoding the enzymes were transformed into *A.* *thaliana*. Arabidopsis is an ideal model plant for testing such activity because monolignol conjugates have not been detected in its native lignin. The substrates needed for monolignol conjugate production are present, however, as aromatic acid CoA intermediates in the lignin biosynthetic pathway and the monolignols are the monomer subunits for lignin polymerization (Smith et al., 2015, 2017). The production of monolignol conjugates by one of the putative FMT or PMT enzymes will therefore generate novel, readily detectable compounds in the Arabidopsis lignin. *SbFMT*, *PvFMT*, *ZmFMT*, *SbPMT*, and *PvPMT* were cloned into a vector with a strong constitutive Arabidopsis ubiquitin 10 promoter to ensure that the genes and enzymes are widely and strongly expressed, along with a C-terminal GFP tag that was used as a proxy for protein expression (*pUBQ10*::*MT-GFP*). The roots and stems of transgenic Arabidopsis were examined for GFP expression. All the protein-GFP fusions showed cytosolic localization, as expected for the acyltransferases based on previous studies (Supplemental Figure S6; Fujiwara et al., 1998; Yu et al., 2008; Panikashvili et al., 2009; Serra et al., 2010; Rautengarten et al., 2012; Wu et al., 2018) and consistent with the cytosolic localization of lignin monomer biosynthesis (Vanholme et al., 2010). The transgenic plants appeared phenotypically identical to wild-type Arabidopsis plants with respect to growth, plant height, and fertility (Supplemental Figure S6). This indicates that the production of new compounds and their integration into the cell wall does not negatively impact plant growth and development.

To further test the activity of the putative FMT and PMT enzymes as feruloyl-CoA or *p*-coumaroyl-CoA monolignol transferases, the incorporation of monolignol conjugates into the lignin polymer was examined. For this analysis, DFRC was performed on alcohol-insoluble residue (AIR) to characterize cell-wall-bound components. DFRC cleaves specific bonds (β-*O*-4 alkyl–aryl ethers) found in lignin, and the products of the assay are representative of the lignin composition (Lu and Ralph, 1997). The ester bonds that are characteristic of monolignol conjugates remain intact through the DFRC process, generating a distinct chemical fingerprint for the presence of conjugates in the lignin polymer (Lu and Ralph, 1997; Regner et al., 2018). The transgenic Arabidopsis expressing *SbFMT, PvFMT*, and *ZmFMT* all produced varying levels of monolignol ferulates (Table 4). These FMTs also produced *p*CA and integrated it into the lignin, based on its release through DFRC (Table 4). This result indicates that the identified grass FMTs are not only acting as FMT enzymes in vitro, but also in planta, and that the FMTs have the same promiscuity for CoA substrates in planta as detected in the enzyme activity assays. The transgenic Arabidopsis plants expressing *SbPMT* yielded a functional SbPMT enzyme that produced monolignol *p*-coumarate conjugates that were integrated into the lignin (Table 4). The transgenic plants expressing *PvPMT* similarly produced monolignol *p*-coumarate conjugates that were integrated into the lignin*.* The variability in the concentration of monolignol conjugates released by DFRC was most likely the result of needing to pool Arabidopsis plants to yield enough sample for analysis.

**Table 4:** Concentrations of (acetylated) monolignol ferulates (FA) and monolignol *p*-coumarates (*p*CA) released by DFRC from the transgenic Arabidopsis whole cell wall stem tissue

DFRC	*p*CA (mg/g whole cell wall) ± stderror	FA (mg/g whole cell wall) ± stderror
Col WT	ND	ND
pUBC::OsFMT-GFP	0.08 ± 0.006^*^	0.67 ± 0.37^*^
pUBC::SbFMT-GFP	0.09 ± 0.03^*^	0.65 ± 0.12^*^
pUBC::PvFMT-GFP	0.03 ± 0.005^*^	0.67 ± 0.56
pUBC::ZmFMT-GFP	0.01 ± 0.005^*^	0.15 ± 0.04^*^
pUBC::SbPMT-GFP	0.42 ± 0.1^*^	ND
pUBC::PvPMT-GFP	1.62 ± 0.4^*^	ND

Values are the average of three to five biological replicates (each with two technical replicates) and the error bars represent standard error.

* indicates *P* < 0.05, as determined by one-sample *t* test (ND indicates product not detected).

The DFRC results demonstrate that the monolignol conjugates are integrated into the lignin polymer. However, in some plant species *p*-coumarate and ferulate are attached to polysaccharides, as found in grasses, sugar beet (*Beta vulgaris*), and spinach (*Spinacia oleracea*) (Ishii, 1997; Scheller and Ulvskov, 2010), cutin (Rautengarten et al., 2012), and suberin (Cheng et al., 2013). Previously identified monolignol transferases, including OsPMT, have been classified as lignin-specific, whereas other transferases, such as rice acyltransferase 10 (OsAT10), are polysaccharide-specific (Bartley et al., 2013). To determine if the identified FMT and PMT enzymes are lignin-specific, mild acidolysis was performed on whole cell wall acid insoluble residue (Lapierre et al., 2018, 2019; Eugene et al., 2020). Mild acidolysis is therefore an orthogonal process to DFRC, using mild acidic conditions to release arabinosyl units from arabinoxylans, keeping the *p*-coumarates and ferulates esters largely intact, i.e. releasing for analysis methyl 5-*O*-*p*-coumaroyl or 5-*O*-feruloyl arabinosides diagnostic of their origin on arabinoxylans. No *p*-coumarates, ferulates, or arabinose-ferulates were detected following mild acidolysis treatment, indicating that the identified FMT and PMT enzymes do not form arabinose conjugates integrated into the cell wall hemicelluloses.

## Discussion and conclusions

Monolignol conjugates comprise a significant proportion of the lignin polymer in grasses and identifying the enzymes responsible for their synthesis could facilitate lignin engineering. However, the BAHD acyltransferases involved in the production of monolignol conjugates in species such as maize, sorghum, and switchgrass were unknown. Five additional monolignol transferases were identified here, including *Zm*FMT, *Sb*FMT, *Sb*PMT, *Pv*FMT, and *Pv*PMT. We characterized the substrate preferences for the enzymes in vitro and confirmed the activity of the enzymes in Arabidopsis. All the enzymes were found to function as feruloyl-CoA monolignol transferases and/or *p*-coumaroyl-CoA monolignol transferases.

The FMT and PMT enzymes from sorghum, switchgrass, and maize were predicted based on their sequence similarity to previously characterized FMT and PMT enzymes, such as OsFMT and OsPMT. There was low sequence similarity between FMT and PMT enzymes, but >60% sequence identity among the grass FMTs or PMTs. As few monolignol transferases have been identified to date, other well-characterized families of BAHD acyltransferases that use similar substrates can be used as a proxy for the extent of sequence similarity within an enzyme family. For example, HCT enzymes are involved in lignin biosynthesis, performing the coupling of *p*-coumaroyl-CoA to shikimate to generate *p*-coumaroyl shikimate. These enzymes, like the grass monolignol transferases that have been discovered to date, are grouped in Clade V of the BAHD acyltransferases (Bontpart et al., 2015). HCTs have been identified in many model plant species and potential biofuel crops, outlining a large family of well-characterized enzymes. The first HCT enzymes were identified in tobacco (*Nicotiana tabacum*) and Arabidopsis (Hoffmann et al., 2003; Hoffmann et al., 2004). Based on sequence homology, HCTs have since been identified in coffee (*Coffea canephora*), coleus (*Coleus blumei*), pine (*Pinus radiata*), poplar, switchgrass, alfalfa (*Medicago sativa*), rice, and sorghum, as well as more ancestral branches of the plant kingdom such as mosses, liverworts, and lycophytes (Chen et al., 2006; Tsai et al., 2006; Lepelley et al., 2007; Shadle et al., 2007; Wagner et al., 2007; Sander and Petersen, 2011; Kim et al., 2012; Escamilla-Treviño et al., 2014; Eudes et al., 2016b; Walker et al., 2016; Wu et al., 2018). The identification of HCTs from such a wide range of species throughout the plant kingdom inspires confidence in using sequence identity to mine plant genomes for more monolignol transferase enzymes, provided they are conserved within certain plant clades. Unlike HCT enzymes, which appear to be conserved across the plant kingdom, there is low sequence identity between AsFMT and the grass FMTs, that is the level of identity is no greater than that observed for any given BAHD within the enzyme family. It is therefore possible that FMT enzymes in eudicots and grasses arose through convergent evolution (Karlen et al., 2016). This has been observed in acyltransferases involved in anthocyanin biosynthesis in *A.* *thaliana* (Luo et al., 2007). Malonyltransferases in Rosid and Asterid eudicot clades had low sequence similarity and were proposed to have evolved the same function convergently (Luo et al., 2007). The differences in catalytic properties observed in these enzymes supported this hypothesis. We observed slight differences in substrate specificity between AsFMT and the grass FMTs, which might reflect independent origins of their FMTs. To this end, sequence similarity alone should not be the only consideration when uncovering future monolignol transferases; rather, the expression pattern of the genes, the broader substrate availability to the enzyme, the evolutionary origin of the enzymes, and the structure of the enzyme should also be considered.

Another important achievement of this research approach was uncovering that the substrate specificity or promiscuity of PMT and FMT enzymes was consistent between the in vitro activity assays and in planta analyses. The FMT enzymes produced significant and detectable amounts of monolignol ferulates and *p*-coumarates in the competition enzyme assays, and transgenic Arabidopsis plants expressing FMTs had lignin-bound monolignol ferulates and *p*-coumarates. The PMT enzymes produced only monolignol *p*-coumarates in the competition enzyme assays and transgenic PMT-expressing plants also only released monolignol *p*-coumarates from the lignin. This observation further confirms the validity of determining BAHD enzyme activity using enzymes produced by cell-free translation and in vitro-synthesized substrates. However, the promiscuity of the FMT enzymes highlights the need to confirm in planta activity, as the true enzyme substrates might have been omitted from those screened in the in vitro assays.

In this study, we focused on a limited array of donor and acceptor substrates specific to monolignol transferase activity. However, there is a large range of other possible substrates for BAHD acyltransferases. Some acyltransferases appear have strong preferences for a small number of substrates (Grienenberger et al., 2009; Kosma et al., 2012; Withers et al., 2012), whereas others have a much broader spectrum of donor and acceptor substrates and may be involved in the production of a larger array of products. For example, LaAT1 from lavender (*Lavandula angustifolia*) and HCT from coleus can use a variety of CoA donors and amine or alcohol acceptors to form ester- or amide-linked end-products (Landmann et al., 2011; Sander and Petersen, 2011). Eudes et al. (2016b) took advantage of the substrate promiscuity of switchgrass and Arabidopsis HCT enzymes to produce valuable hydroxycinnamate and benzoate metabolites for lignin engineering. Together, these data seem to indicate that BAHD acyltransferases may be capable of utilizing a broad array of substrates even beyond those biologically available, offering possibilities for novel biosynthesis via cell-free enzymatic approaches. It is also possible that the monocot transferases characterized in this study could have a broader range of substrates than those in our current focus on the biosynthesis of monolignol conjugates incorporated into cell walls. For example, it is possible that other substrates may be used by these enzymes, leading to additional soluble phenolic products. There may also be other FMT and PMT enzymes in sorghum, switchgrass, and maize with different activities and/or different spatio-temporal localizations (Withers et al., 2012; Karlen et al., 2016). Further examination of the whole family of FMT and PMT enzymes in each species could reveal important information about the evolution and breadth of function of this family of proteins.

As we uncovered these monolignol transferases and elucidated their enzymatic activities, several intriguing trends emerged from the data. (1) FMT enzymes are more promiscuous than the PMT enzymes; presumably due to a larger binding site required to accommodate the methoxy group of the feruloyl-CoA; (2) PMT enzymes are fairly specific for *p*-coumaroyl-CoA; (3) all three monolignols bind efficiently in the enzyme binding sites, implying few restrictive contacts with the methoxy groups of either the G (coniferyl alcohol) or S (sinapyl alcohol) substrates; and (4) the Brachypodium FMT enzyme showed that enzymes that have a high degree of sequence identity to known FMTs or PMTs do not necessarily exhibit the same enzyme activity under the current assay conditions. Although crystal structures have already been elucidated for a number of BAHD acyltransferases, including vinorine synthase (Ma et al., 2005), and HCTs from sorghum and switchgrass (Walker et al., 2013; Eudes et al., 2016b), homology models have not yet provided definitive insights.

The discovery of additional BAHD enzymes that function as monolignol transferases in vitro and in planta presents new opportunities, especially with respect to the production of optimal biofuels and bioproducts. These transferases are from potential biofuel crops and could be important in manipulating the levels of monolignol *p*-coumarates and/or monolignol ferulates in these species to optimize production of “clip-off” products (Timokhin et al., 2020) or improve cell wall digestibility.

## Materials and methods

### Selection of gene sequences

Gene sequences were obtained from NCBI GenBank and were selected by their sequence identity with monolignol acyltransferases, especially from rice (*O.* *sativa*, *OsAT4/OsPMT*, and *OsAT5/OsFMT*) (Withers et al., 2012; Karlen et al., 2016). Genes from the grasses (sorghum [*S.* *bicolor*], switchgrass [*P.* *virgatum*], Brachypodium [*B.* *distachyon*], maize [*Z.* *mays*], and rice [*O.* *sativa*]) were prepared along with the *Angelica sinensis AsFMT*, see (Table 1). Genbank or accession numbers for the genes of interest are as follows: XM_017385232.1 (*AsFMT*), XM_015785190.2 (*OsFMT*), XM_015765814.2 (*OsPMT*), XM_002441921.2 (*SbFMT*), XM_002439193.2 (*SbPMT*), Pavir.3KG495300 (PAC:41613500) (in Phytozome *P.* *virgatum* v5.1; *PvFMT*), XM_039943249.1 (*PvPMT*), and Zm00001d035246_T001 (in Phytozome *Z.* *mays* RefGen_V4; *ZmFMT*) (Goodstein et al., 2012). Protein sequence comparisons were made with NCBI BLAST+ 2.5.0 using default settings. The sequence identity is reported both as a percentage identity over the matched regions, as well as a weighted “coverage” statistic, in which the numerator is the number of identical residues, and the denominator is the length of the matched region.

### Cloning vector

Genes were synthesized by GenScript Corporation (Piscataway, NJ) and cloned into the wheatgerm cell-free expression vector, pEU (Sawasaki et al., 2000), which contains an SP6 promoter and omega enhancer sequence from tobacco mosaic virus. Plasmid DNA was purified from *Escherichia coli* using a commercial purification kit, treated with proteinase K and then re-purified to remove residual RNAse activity and to concentrate the DNA.

### Transcription

Messenger RNA was prepared by adding 12.8 U of SP6 RNA polymerase and 6.4 U of RNasin RNase inhibitor (Promega Corporation, Madison, WI) to plasmid DNA (0.2 mg/mL or higher) in the presence of 2.5 mM each of UTP, CTP, ATP, and GTP and 20 mM magnesium acetate, 2 mM spermidine hydrochloride, 10 mM dithiothreitol (DTT), and 80 mM HEPES-KOH, pH 7.8. Transcription reactions were incubated at 37°C for 4 h.

### Cell-free translation

Enzymes were produced using a wheatgerm cell-free translation bilayer method previously reported (Makino et al., 2014; Takasuka et al., 2014). Briefly, a translation reaction mix was combined with the transcription at a ratio of 4:1 such that final reagent concentrations were 25% (v/v) wheatgerm extract 2240 (CellFree Sciences, Matsuyama, Japan), 29 mM HEPES-KOH, 55 mM potassium acetate, 5.4 mM magnesium acetate, 0.6 mM spermidine HCl, 4 mM DTT, 0.7 mM ATP, 0.14 mM GTP, 8.8 mM creatine phosphate, 0.04 mg/mL creatine kinase, 0.003% sodium azide (w/v), 0.3 mM each amino acid, pH 7.8. A 125-µL amino-acid-containing feeding layer was first added to a U-bottom 96-well plate chamber, then 25 µL of the denser reaction mixture was underlaid. The plate was sealed and incubated at 22°C for 18 h. The fully diffused 150 µL bilayer reaction was then harvested and analyzed for protein expression by SDS-PAGE. Translated proteins were quantified by BioRad stain-free analysis as previously reported (Makino et al., 2014).

### Synthesis of substrates for enzyme assays

Feruloyl-CoA and *p*-coumaroyl-CoA were synthesized using the 4-coumarate-CoA ligase (4CL-1) enzyme from tobacco (*N.* *tabacum*) (Beuerle and Pichersky, 2002). To a 10-mL solution of 50 mM Tris–HCl pH 8 and 2.5 mM MgCl_2_, 3.3 mg of ferulic acid or *p*-coumaric acid, 2 mg of free CoA, and 6.9 mg of ATP were added. The reactions were started by adding 0.25 mg of purified Nt4CL-1. Following a 5-h incubation at room temperature, another 2 mg of free CoA acid, 6.9 mg of ATP salt, and 0.25 mg of Nt4CL-1 were added to each reaction. The reactions were allowed to proceed overnight at room temperature. The buffer was adjusted by the addition of ammonium acetate to make a 4% ammonium acetate solution (0.4 g per reaction). The reaction mixtures were loaded onto a C18 silica column (RediSep Rf Gold, Teledyne) pre-conditioned with 5 volumes of methanol, 5 volumes of ddH_2_O and 5 columns of 4% ammonium acetate. The columns were washed with 4% ammonium acetate until the free CoA and excess free acid was absent based on UV spectrophotometry (absorbance at 340 nm, A_340_). The CoA products (feruloyl-CoA or *p*-coumaroyl-CoA) were then eluted with ddH_2_O and monitored by UV spectroscopy at A_333_ for *p*-coumaroyl-CoA and A_345_ for feruloyl-CoA. The product concentration was calculated from an aliquot of the final solution diluted with methanol (1:1, v/v) using the equation:

M (concentration)=(maximum absorbance*dilution factor)/extinction coefficient

and an extinction coefficient of 21,000 L mol^−1^ cm^−1^ for *p*-coumaroyl-CoA (Stockigt and Zenk, 1975) and 19,000 L mol^−1^ cm^−1^ for feruloyl-CoA (Gross and Zenk, 1966). The products were lyophilized and resuspended in autoclaved ddH_2_O to a concentration of 20–30 mg/mL and stored at –80°C.

The monolignols (*p*-coumaryl [H], coniferyl [G], and sinapyl [S] alcohols) and monolignol conjugate products (ML-*p*CA and ML-FA) were prepared as previously described (Zhu et al., 2013). The calibration and internal standards for the DFRC assay were synthesized from *p*-coumaric and ferulic acid as previously described (Regner et al., 2018).

### Activity screening

Transferases were screened for activity with feruloyl-CoA and *p*-coumaroyl-CoA and all three monolignols (*p*-coumaryl, coniferyl, and sinapyl alcohol) alongside positive (OsPMT or OsFMT) and negative controls (no enzyme) following the procedure previously reported (Withers et al., 2012). As *Os*PMT was used as one of the positive control enzymes and its optimal enzyme activity conditions were described in Withers et al. (2012), the same conditions were used for all the putative and control acyltransferase enzymes. Briefly, the assay was initiated by adding 10 µL of the cell-free translation reaction containing one of the PMT or FMT enzymes at a concentration of 1.5–2 µM to a reaction containing, at a final concentration, 50 mM sodium phosphate buffer at pH 6, 1 mM DTT, 1 mM CoA thioester, 1 mM monolignol mixture (*p*-coumaryl alcohol, coniferyl alcohol and sinapyl alcohol, each at 1 mM final concentration), and deionized water in a final volume of 50 µL. After a 30-min incubation, the reaction was stopped by the addition of an equal volume of 100 mM hydrochloric acid. Reaction products were solubilized by adjusting the solution to 50% methanol. An identical assay with no enzyme added was performed for every reaction. Samples were filtered through 0.2 µm filters prior to analysis by LC-MS.

The filtered product solutions were analyzed by LC-MRM-MS (Shimadzu Prominence LC equipped with a photodiode array [PDA] and TQ8040 mass spectrometer). Products were run through a Kinetex XB-C18 column (100 Å, 250 × 4.60 mm; Phenomenex), using water (Solvent A) and methanol (Solvent B) as solvents. The LCMS method had an initial Solvent B concentration of 5%, followed by a linear increase to 100% Solvent B over 30 min, with a hold for 4 min, followed by a return to 5% over 1 min, held for 10 min, with a flow rate of 1 mL/min. Compounds were detected using PDA wavelengths of 250–400 nm and Q3 scans in negative-ion and positive-ion mode between 120 and 600 *m*/*z*. Products were verified by matching retention times and mass spectrometric fragmentation patterns with those from authentic standards.

Enzyme activity measurements were determined by running enzyme assays as described above, with a CoA thioester range of 0.2–1.0 mM. Reactions were started with the addition of 0.15 µg of cell-free enzyme, as determined by stain-free gel analysis (Supplemental Figure S4). After 60 min, the reaction was stopped and 7.5 µg 4-methyl catechol was added to each reaction as an internal standard. This time point was selected because it was sufficient for the reactions to have reached saturation. The product area of the monolignol conjugates was determined using PDA data and was normalized to the internal standard peak area. The normalized product area was then expressed as product area per µg of acyltransferase enzyme.

### Construction of plant transformation vectors, transformation into Arabidopsis, and Arabidopsis growth conditions

Gateway cloning technology (Invitrogen) was used to generate the *ProUBQ10*::*AsFMT-GFP*, *ProUBQ10*::*OsFMT-GFP*, *ProUBQ10*::*SbFMT-GFP*, *ProUBQ10*::*PvFMT-GFP*, *ProUBQ10*::*ZmFMT-GFP*, *ProUBQ10*::*SbPMT-GFP*, and *ProUBQ10*::*PvPMT-GFP* constructs (*pUBC*:*GFP* vector; Grefen et al., 2010; primers are listed in Supplemental Table S1). The plant acyltransferase constructs were introduced into *Agrobacterium tumefaciens* strain GV3101 and transformed into Arabidopsis (*A.* *thaliana*) Col-0 using the floral dip method (Clough and Bent, 1998) to generate transgenic plants.

Wild-type and transgenic Arabidopsis seeds were plated on media with half-strength Murashige and Skoog media (Sigma–Aldrich) and, for the plates with transgenic seeds, glufosinate antibiotic. Seedlings grew on plates for 2 weeks and then were transplanted to soil. Transgenics were selected based on resistance to Basta. *Arabidopsis thaliana* plants were grown in a growth chamber under long day conditions (16-h light/8-h dark) at 21°C. When the plants were 2–3 weeks old, genomic DNA was extracted from leaves for genotyping using primers listed in Supplemental Table S1.

### Imaging of transgenic plants

Small segments of mature stems (2 months old) were cut and sectioned longitudinally into distilled water. The fresh sections were then imaged using a Zeiss 710 confocal microscope using a 488 nm laser to collect GFP data and chlorophyll autofluorescence (laser power 7.0, gain 850, collection bandwidth 493–552 nm, pinhole 1 Airy Unit = 1.7 µm section). Images were pseudo-colored green and red for GFP and chlorophyll signals, respectively.

### Cell wall analysis of transgenic plants

DFRC was performed as previously described (Regner et al., 2018). Briefly, 30–50 mg of ground and solvent-extracted whole cell wall material was subjected to DFRC analysis. The internal standard mixture for each sample contained d_8_-CA, d_8_-SA, d_8_-S*p*HBA, d_10_-SDD*p*CA, and d_10_ SDDFA. Statistical analysis was performed using a one-sample t-test to compare WT and transgenic lines, *P* < 0.05. Mild acidolysis was performed as described in Eugene et al. (2020). DFRC and mild acidolysis results were expressed as the average of three to five biological replicates per transgenic line. Each biological replicate was composed of a pool of 15 Arabidopsis stems, with siliques and leaves removed.

## Accession numbers

Genbank or accession numbers for the genes of interest are as follows: XM_017385232.1(*AsFMT*), XM_015785190.2 (*OsFMT*), XM_015765814.2 (*OsPMT*), XM_002441921.2 (*SbFMT*), XM_002439193.2 (*SbPMT*), Pavir.3KG495300 (PAC:41613500) (in Phytozome *P.* *virgatum* v5.1; *PvFMT*), XM_039943249.1 (*PvPMT*), and Zm00001d035246_T001 (in Phytozome *Z.* *mays* RefGen_V4; *ZmFMT*).

## Supplemental data

The following materials are available in the online version of this article.

**Supplemental Figure S1.** Denaturing gels showing cell-free protein synthesis of acyltransferases and analysis of translation results.

**Supplemental Figure S2.** Individual enzyme assays with three monolignol acceptors (*p*-coumaryl alcohol [H], coniferyl alcohol [G], and sinapyl alcohol [S]) and feruloyl-CoA as a donor.

**Supplemental Figure S3.** Individual enzyme assays with three monolignol acceptors (*p*-coumaryl alcohol [H], coniferyl alcohol [G], and sinapyl alcohol [S]) and *p*-coumaroyl-CoA as a donor.

**Supplemental Figure S4.** BioRad Stain-Free denaturing gel analyses of acyltransferases translation reactions used to produce enzyme needed carry out activity measurements.

**Supplemental Figure S5.** Enzyme activity for the FMT and PMT enzymes.

**Supplemental Figure S6.** Growth and localization of acyltransferase transgenic lines.

**Supplemental Table S1.** Cloning and genotyping primers, and full coding sequences for FMT and PMT genes of interest.

## Supplementary Material

kiac035_Supplementary_Data

## Notes

R.A.S., E.T.B., C.A.B., S.D.K., J.R., and B.G.F. conceived the research plans and identified the enzymes of interest. E.T.B. and K.V.M. generated the enzymes. J.R. and B.G.F. supervised the research. R.A.S. and S.D.K. performed the enzyme assays. R.A.S. performed the plant transformation, microscopy, and DFRC analysis. A.E. performed the mild acidolysis analysis. A.J.S. provided technical assistance to R.A.S. R.A.S. wrote the article with contributions from all the authors and agrees to serve as the author responsible for contact and ensures communication.

The author responsible for distribution of materials integral to the findings presented in this article in accordance with the policy described in the Instructions for Authors (https://academic.oup.com/plphys/pages/General-Instructions) is Rebecca Smith (rasmith29@wisc.edu).

## References

[kiac035-B1] Bartley LE , Peck ML, Kim SR, Ebert B, Manisseri C, Chiniquy DM, Sykes R, Gao L, Rautengarten C, Vega-Sanchez ME, et al (2013) Overexpression of a BAHD acyltransferase, OsAt10, alters rice cell wall hydroxycinnamic acid content and saccharification. Plant Physiol 161: 1615–1633 23391577 10.1104/pp.112.208694 PMC3613443

[kiac035-B2] Beekwilder J , Alvarez-Huerta M, Neef E, Verstappen FW, Bouwmeester HJ, Aharoni A (2004) Functional characterization of enzymes forming volatile esters from strawberry and banana. Plant Physiol 135: 1865–1878 15326278 10.1104/pp.104.042580 PMC520758

[kiac035-B3] Beuerle T , Pichersky E (2002) Enzymatic synthesis and purification of aromatic coenzyme a esters. Anal Biochem 302: 305–312 11878812 10.1006/abio.2001.5574

[kiac035-B4] Bhalla A , Bansal N, Pattathil S, Li M, Shen W, Particka CA, Semaan R, Gonzales-Vigil E, Karlen SD, Ralph J, et al (2018) Engineered lignin in poplar biomass facilitates Cu-AHP pretreatment. ACS Sustain Chem Eng 6: 2932–2941

[kiac035-B5] Bontpart T , Cheynier V, Ageorges A, Terrier N (2015) BAHD or SCPL acyltransferase? What a dilemma for acylation in the world of plant phenolic compounds. New Phytol 208: 695–707 26053460 10.1111/nph.13498

[kiac035-B6] Chen F , Reddy MSS, Temple S, Jackson L, Shadle G, Dixon RA (2006) Multi-site genetic modulation of monolignol biosynthesis suggests new routes for formation of syringyl lignin and wall-bound ferulic acid in alfalfa (*Medicago sativa* L.). Plant J 48: 113–124 16972868 10.1111/j.1365-313X.2006.02857.x

[kiac035-B7] Cheng AX , Gou JY, Yu XH, Yang H, Fang X, Chen XY, Liu CJ (2013) Characterization and ectopic expression of a populus hydroxyacid hydroxycinnamoyltransferase. Mol Plant 6: 1889–1903 23709341 10.1093/mp/sst085

[kiac035-B8] Clough SJ , Bent AF (1998) Floral dip: a simplified method for *Agrobacterium*-mediated transformation of *Arabidopsis thaliana*. Plant J 16: 735–743 10069079 10.1046/j.1365-313x.1998.00343.x

[kiac035-B9] D’Auria JC (2006) Acyltransferases in plants: A good time to be BAHD. Curr Opin Plant Biol 9: 331–340 16616872 10.1016/j.pbi.2006.03.016

[kiac035-B10] Dima O , Morreel K, Vanholme B, Kim H, Ralph J, Boerjan W (2015) Small glycosylated lignin polymers are stored in Arabidopsis leaf vacuoles. Plant Cell 27: 695–710 25700483 10.1105/tpc.114.134643 PMC4558659

[kiac035-B11] Escamilla-Treviño LL , Shen H, Hernandez T, Yin Y, Xu Y, Dixon RA (2014) Early lignin pathway enzymes and routes to chlorogenic acid in switchgrass (*Panicum virgatum* L.). Plant Mol Biol 84: 565–576 24190737 10.1007/s11103-013-0152-y

[kiac035-B12] Eudes A , Mouille M, Robinson DS, Benites VT, Wang G, Roux L, Tsai YL, Baidoo EE, Chiu TY, Heazlewood JL, et al (2016a) Exploiting members of the BAHD acyltransferase family to synthesize multiple hydroxycinnamate and benzoate conjugates in yeast. Microb Cell Factor 15: 198 10.1186/s12934-016-0593-5 PMC5117604 27871334

[kiac035-B13] Eudes A , Pereira JH, Yogiswara S, Wang G, Teixeira Benites V, Baidoo EE, Lee TS, Adams PD, Keasling JD, Loque D (2016b) Exploiting the substrate promiscuity of hydroxycinnamoyl-CoA:shikimate hydroxycinnamoyl transferase to reduce lignin. Plant Cell Physiol 57: 568–579 26858288 10.1093/pcp/pcw016 PMC4790474

[kiac035-B14] Eugene A , Lapierre C, Ralph J (2020) Improved analysis of arabinoxylan-bound hydroxycinnamate conjugates in grass cell walls. Biotechnol Biofuels 13: 201–207 33303001 10.1186/s13068-020-01841-6 PMC7731738

[kiac035-B15] Fujiwara H , Tanaka Y, Yonekura-Sakakibara K, Fukuchi-Mizutani M, Nakao M, Fukui Y, Yamaguchi M, Ashikari T, Kusumi T (1998) cDNA cloning, gene expression and subcellular localization of anthocyanin 5-aromatic acyltransferase from *Gentiana triflora*. Plant J 16: 421–431 9881162 10.1046/j.1365-313x.1998.00312.x

[kiac035-B74] Goodstein DM , Shu SQ, Howson R, Neupane R, Hayes RD, Fazo J, Mitros T, Dirks W, Hellsten U, Putnam N, Rokhsar DS (2012) Phytozome: a comparative platform for green plant genomics. Nucleic Acids Res 40: D1178–D1186 22110026 10.1093/nar/gkr944 PMC3245001

[kiac035-B16] Grabber JH , Quideau S, Ralph J (1996) *p*-Coumaroylated syringyl units in maize lignin; implications for β-ether cleavage by thioacidolysis. Phytochemistry 43: 1189–1194

[kiac035-B17] Grabber JH , Ralph J, Hatfield RD (2002) Model studies of ferulate-coniferyl alcohol cross-product formation in primary maize walls: Implications for lignification in grasses. J Agr Food Chem 50: 6008–6016 12358473 10.1021/jf0205312

[kiac035-B18] Grefen C , Donald N, Hashimoto K, Kudla J, Schumacher K, Blatt MR (2010) A ubiquitin-10 promoter-based vector set for fluorescent protein tagging facilitates temporal stability and native protein distribution in transient and stable expression studies. Plant J 64: 355–365 20735773 10.1111/j.1365-313X.2010.04322.x

[kiac035-B19] Grienenberger E , Besseau S, Geoffroy P, Debayle D, Heintz D, Lapierre C, Pollet B, Heitz T, Legrand M (2009) A BAHD acyltransferase is expressed in the tapetum of Arabidopsis anthers and is involved in the synthesis of hydroxycinnamoyl spermidines. Plant J 58: 246–259 19077165 10.1111/j.1365-313X.2008.03773.x

[kiac035-B20] Gross GG , Zenk MH (1966) Darstellung und eigenschaften von coenzym a-thiolestern substituierter zimtsäuren [Preparation and properties of coenzyme A thioesters of substituted cinnamic acids]. Z Naturforsch B B21: 683–690

[kiac035-B21] Hatfield RD , Marita JM, Frost K, Grabber JH, Lu F, Kim H, Ralph J (2009) Grass lignin acylation: *p*-coumaroyl transferase activity and cell wall characteristics of C3 and C4 grasses. Planta 229: 1253–1267 19288269 10.1007/s00425-009-0900-z

[kiac035-B22] Hoffmann L , Besseau S, Geoffroy P, Ritzenthaler C, Meyer D, Lapierre C, Pollet B, Legrand M (2004) Silencing of hydroxycinnamoyl-coenzyme A shikimate/quinate hydroxycinnamoyltransferase affects phenylpropanoid biosynthesis. Plant Cell 16: 1446–1465 15161961 10.1105/tpc.020297 PMC490038

[kiac035-B23] Hoffmann L , Maury S, Martz F, Geoffroy P, Legrand M (2003) Purification, cloning, and properties of an acyltransferase controlling shikimate and quinate ester intermediates in phenylpropanoid metabolism. J Biol Chem 278: 95–103 12381722 10.1074/jbc.M209362200

[kiac035-B24] Ishii T (1997) Structure and functions of feruloylated polysaccharides. Plant Sci 127: 111–127

[kiac035-B25] Kaal J , Serrano O, del Río JC, Rencoret J (2018) Radically different lignin composition in *Posidonia* species may link to differences in organic carbon sequestration capacity. Org Geochem 124: 247–256

[kiac035-B26] Karlen SD , Fasahati P, Mazaheri M, Serate J, Smith RA, Sirobhushanam S, Chen M, Tymokhin V, Cass CL, Liu S, et al (2020) Assessing the viability of recovering hydroxycinnamic acids from lignocellulosic biorefinery alkaline pretreatment waste streams. ChemSusChem 13: 2012–2024 31984673 10.1002/cssc.201903345 PMC7217007

[kiac035-B27] Karlen SD , Free HCA, Padmakshan D, Smith BG, Ralph J, Harris PJ (2018) Commelinid monocotyledon lignins are acylated by *p*-coumarate. Plant Physiol 177: 513–521 29724771 10.1104/pp.18.00298 PMC6001335

[kiac035-B28] Karlen SD , Zhang C, Peck ML, Smith RA, Padmakshan D, Helmich KE, Free HCA, Lee S, Smith BG, Lu F, et al (2016) Monolignol ferulate conjugates are naturally incorporated into plant lignins. Sci Adv 2: e1600393 27757415 10.1126/sciadv.1600393 PMC5065250

[kiac035-B29] Kim IA , Kim BG, Kim M, Ahn JH (2012) Characterization of hydroxycinnamoyltransferase from rice and its application for biological synthesis of hydroxycinnamoyl glycerols. Phytochemistry 76: 25–31 22285622 10.1016/j.phytochem.2011.12.015

[kiac035-B30] Kim KH , Dutta T, Ralph J, Mansfield SD, Simmons BA, Singh S (2017) Impact of lignin polymer backbone esters on ionic liquid pretreatment of poplar. Biotechnol Biofuels 10: 101 28439294 10.1186/s13068-017-0784-2 PMC5399332

[kiac035-B31] Kosma DK , Molina I, Ohlrogge JB, Pollard M (2012) Identification of an Arabidopsis fatty alcohol: Caffeoyl-coenzyme A acyltransferase required for the synthesis of alkyl hydroxycinnamates in root waxes. Plant Physiol 160: 237–248 22797656 10.1104/pp.112.201822 PMC3440202

[kiac035-B32] Landmann C , Huecherig S, Fink B, Hoffmann T, Dittlein D, Coiner HA, Schwab W (2011) Substrate promiscuity of a rosmarinic acid synthase from lavender (*Lavandula angustifolia* L.). Planta 234: 305–320 21424826 10.1007/s00425-011-1400-5

[kiac035-B33] Lapierre C , Voxeur A, Boutet S, Ralph J (2019) Arabinose conjugates diagnostic of ferulate-ferulate and ferulate-monolignol cross-coupling are released by mild acidolysis of grass cell walls. J Agr Food Chem 67: 12962–12971 31644281 10.1021/acs.jafc.9b05840

[kiac035-B34] Lapierre C , Voxeur A, Karlen SD, Helm RF, Ralph J (2018) Evaluation of feruloylated and *p*-coumaroylated arabinosyl units in grass arabinoxylans by acidolysis in dioxane/methanol. J Agr Food Chem 66: 5418–5424 29763561 10.1021/acs.jafc.8b01618

[kiac035-B35] Lepelley M , Cheminade G, Tremillon N, Simkin A, Caillet V, McCarthy J (2007) Chlorogenic acid synthesis in coffee: An analysis of CGA content and real-time RT-PCR expression of HCT, HQT, C3H1, and CCoAOMT1 genes during grain development in *C. canephora*. Plant Sci 172: 978–996

[kiac035-B36] Lu F , Karlen SD, Regner M, Kim H, Ralph SA, Sun R-C, Kuroda K-I, Augustin MA, Mawson R, Sabarez H, et al (2015) Naturally *p*-hydroxybenzoylated lignins in palms. BioEnergy Res 8: 934–952

[kiac035-B37] Lu F , Ralph J (1997) The DFRC method for lignin analysis. Part 1. A new method for β-aryl ether cleavage: lignin model studies. J Agr Food Chem 45: 4655–4660

[kiac035-B38] Lu F , Ralph J (1999) Detection and determination of *p*-coumaroylated units in lignins. J Agri Food Chem 47: 1988–1992 10.1021/jf981140j 10552483

[kiac035-B39] Luo J , Nishiyama Y, Fuell C, Taguchi G, Elliott K, Hill L, Tanaka Y, Kitayama M, Yamazaki M, Bailey P, et al (2007) Convergent evolution in the BAHD family of acyl transferases: Identification and characterization of anthocyanin acyl transferases from *Arabidopsis thaliana*. Plant J 50: 678–695 17425720 10.1111/j.1365-313X.2007.03079.x

[kiac035-B40] Ma XY , Koepke J, Panjikar S, Fritzsch G, Stockigt J (2005) Crystal structure of vinorine synthase, the first representative of the BAHD superfamily. J Biol Chem 280: 13576–13583 15665331 10.1074/jbc.M414508200

[kiac035-B41] Makino S , Beebe ET, Markley JL, Fox BG (2014) Cell-free protein synthesis for functional and structural studies. Meth Mol Biol 1091: 161–178 10.1007/978-1-62703-691-7_11 24203331

[kiac035-B42] Marita JM , Hatfield RD, Rancour DM, Frost KE (2014) Identification and suppression of the *p*-coumaroyl CoA:hydroxycinnamyl alcohol transferase in *Zea mays* L. Plant J 78: 850–864 24654730 10.1111/tpj.12510 PMC4282748

[kiac035-B43] Panikashvili D , Shi JX, Schreiber L, Aharoni A (2009) The Arabidopsis DCR encoding a soluble BAHD acyltransferase is required for cutin polyester formation and seed hydration properties. Plant Physiol 151: 1773–1789 19828672 10.1104/pp.109.143388 PMC2785978

[kiac035-B44] Petrik DL , Karlen SD, Cass CL, Padmakshan D, Lu F, Liu S, Le Bris P, Antelme S, Santoro N, Wilkerson CG, et al (2014) *p-Coumaroyl*-CoA:Monolignol Transferase (PMT) acts specifically in the lignin biosynthetic pathway in *Brachypodium distachyon*. Plant J 77: 713–726 24372757 10.1111/tpj.12420 PMC4282527

[kiac035-B45] Ralph J (2010) Hydroxycinnamates in lignification. Phytochemistry Rev 9: 65–83

[kiac035-B46] Ralph J , Hatfield RD, Quideau S, Helm RF, Grabber JH, Jung H-JG (1994) Pathway of *p*-coumaric acid incorporation into maize lignin as revealed by NMR. J Am Chem Soc 116: 9448–9456

[kiac035-B47] Rautengarten C , Ebert B, Ouellet M, Nafisi M, Baidoo EEK, Benke P, Stranne M, Mukhopadhyay A, Keasling JD, Sakuragi Y, et al (2012) Arabidopsis deficient in cutin ferulate encodes a transferase required for feruloylation of ω-hydroxy fatty acids in cutin polyester. Plant Physiol 158: 654–665 22158675 10.1104/pp.111.187187 PMC3271757

[kiac035-B48] Regner M , Bartuce A, Padmakshan D, Ralph J, Karlen SD (2018) Reductive cleavage method for quantitation of monolignols and low-abundance monolignol conjugates. ChemSusChem 11: 1600–1605 29603658 10.1002/cssc.201800617 PMC6001451

[kiac035-B49] Rencoret J , Ralph J, Marques G, Gutiérrez A, Martínez ÁT, del Río JC (2013) Structural characterization of the lignin from coconut (*Cocos nucifera*) coir fibers. J Agri Food Chem 61: 2434–2445 10.1021/jf304686x 23398235

[kiac035-B50] Sander M , Petersen M (2011) Distinct substrate specificities and unusual substrate flexibilities of two hydroxycinnamoyltransferases, rosmarinic acid synthase and hydroxycinnamoyl-CoA:shikimate hydroxycinnamoyl-transferase, from *Coleus blumei* Benth. Planta 233: 1157–1171 21318289 10.1007/s00425-011-1367-2

[kiac035-B51] Sawasaki T , Hasegawa Y, Tsuchimochi M, Kasahara Y, Endo Y (2000) Construction of an efficient expression vector for coupled transcription/translation in a wheat germ cell-free system. Nucleic Acids Symp Ser 44: 9–10 10.1093/nass/44.1.9 12903243

[kiac035-B52] Scheller HV , Ulvskov P (2010) Hemicelluloses. Annu Rev Plant Biol, 61: 263–289 20192742 10.1146/annurev-arplant-042809-112315

[kiac035-B53] Serra O , Hohn C, Franke R, Prat S, Molinas M, Figueras M (2010) A feruloyl transferase involved in the biosynthesis of suberin and suberin-associated wax is required for maturation and sealing properties of potato periderm. Plant J 62: 277–290 20088895 10.1111/j.1365-313X.2010.04144.x

[kiac035-B54] Shadle G , Chen F, Reddy MSS, Jackson L, Nakashima J, Dixon RA (2007) Down-regulation of hydroxycinnamoyl CoA: Shikimate hydroxycinnamoyl transferase in transgenic alfalfa affects lignification, development and forage quality. Phytochemistry 68: 1521–1529 17466347 10.1016/j.phytochem.2007.03.022

[kiac035-B55] Sibout R , Le Bris P, Legee F, Cezard L, Renault H, Lapierre C (2016) Structural redesigning Arabidopsis lignins into alkali-soluble lignins through the expression of *p*-coumaroyl-CoA:monolignol transferase PMT. Plant Physiol 170: 1358–1366 26826222 10.1104/pp.15.01877 PMC4775143

[kiac035-B56] Smith RA , Gonzales-Vigil E, Karlen SD, Park J-Y, Lu F, Wilkerson CG, Samuels L, Mansfield SD, Ralph J (2015) Engineering monolignol *p*-coumarate conjugates into Poplar and Arabidopsis lignins. Plant Physiol 169: 2992–3001 26511914 10.1104/pp.15.00815 PMC4677885

[kiac035-B57] Smith RA , Scheutz M, Karlen SD, Bird D, Tokunaga N, Sato Y, Mansfield SD, Ralph J, Samuels AL (2017) Defining the diverse cell populations contributing to lignification in Arabidopsis stems. Plant Physiol 174: 1028–1036 28416705 10.1104/pp.17.00434 PMC5462051

[kiac035-B58] St-Pierre B , De Luca V (2000) Evolution of acyltransferase genes: Origin and diversification of the BAHD superfamily of acyltransferases involved in secondary metabolism. Recent Adv Phytochem 34: 285–315

[kiac035-B59] Stockigt J , Zenk MH (1975) Chemical syntheses and properties of hydroxycinnamoyl-coenzyme A derivatives. Z Naturforsch C C30: 352–358 10.1515/znc-1975-5-609 126581

[kiac035-B60] Takasuka TE , Walker JA, Bergeman LF, Vander Meulen KA, Makino S, Elsen NL, Fox BG (2014) Cell-free translation of biofuel enzymes. Meth Mol Biol 1118: 71–95 10.1007/978-1-62703-782-2_5 PMC5820533 24395410

[kiac035-B61] Timokhin VI , Regner M, Motagamwala AH, Sener C, Karlen SD, Dumesic JA, Ralph J (2020) Production of *p*-coumaric acid from corn GVL-lignin. ACS Sustain Chem Eng 8: 17427–17438

[kiac035-B62] Tsai CJ , Harding SA, Tschaplinski TJ, Lindroth RL, Yuan YN (2006) Genome-wide analysis of the structural genes regulating defense phenylpropanoid metabolism in *Populus*. New Phytol 172: 47–62 16945088 10.1111/j.1469-8137.2006.01798.x

[kiac035-B63] Vanholme R , Demedts B, Morreel K, Ralph J, Boerjan W (2010) Lignin biosynthesis and structure. Plant Physiol 153: 895–905 20472751 10.1104/pp.110.155119 PMC2899938

[kiac035-B64] Vermaas JV , Dixon RA, Chen F, Mansfield SD, Boerjan W, Ralph J, Crowley MF, Beckham GT (2019) Passive membrane transport of lignin-related compounds. Proc Natl Acad Sci USA 116: 23117–23123 31659054 10.1073/pnas.1904643116 PMC6859372

[kiac035-B65] Wagner A , Ralph J, Akiyama T, Flint H, Phillips L, Torr KM, Nanayakkara B, Te Kiri L (2007) Exploring lignification in conifers by silencing hydroxycinnamoyl-CoA:shikimate hydroxycinnamoyltransferase in *Pinus radiata*. Proc Natl Acad Sci, USA 104: 11856–11861 17609384 10.1073/pnas.0701428104 PMC1913891

[kiac035-B66] Walker AM , Hayes RP, Youn B, Vermerris W, Sattler SE, Kang C (2013) Elucidation of the structure and reaction mechanism of sorghum hydroxycinnamoyltransferase and its structural relationship to other coenzyme A-dependent transferases and synthases. Plant Physiol 162: 640–651 23624856 10.1104/pp.113.217836 PMC3668059

[kiac035-B67] Walker AM , Sattler SA, Regner M, Jones JP, Ralph J, Vermerris W, Sattler SE, Kang C (2016) The structure and catalytic mechanism of *Sorghum bicolor* caffeoyl-CoA *O*-methyltransferase. Plant Physiol 172: 78–92 27457122 10.1104/pp.16.00845 PMC5074638

[kiac035-B68] Wilkerson CG , Mansfield SD, Lu F, Withers S, Park J-Y, Karlen SD, Gonzales-Vigil E, Padmakshan D, Unda F, Rencoret J, et al (2014) Monolignol ferulate transferase introduces chemically labile linkages into the lignin backbone. Science 344: 90–93 24700858 10.1126/science.1250161

[kiac035-B69] Withers S , Lu F, Kim H, Zhu Y, Ralph J, Wilkerson CG (2012) Identification of a grass-specific enzyme that acylates monolignols with *p*-coumarate. J Biol Chem 287: 8347–8355 22267741 10.1074/jbc.M111.284497 PMC3318722

[kiac035-B70] Wu YF , Zhao Y, Liu XY, Gao S, Cheng AX, Lou HX (2018) Isolation and functional characterization of hydroxycinnamoyltransferases from the liverworts *Plagiochasma appendiculaturn* and *Marchantia paleacea*. Plant Physiol Biochem 129: 400–410 30691636 10.1016/j.plaphy.2018.06.019

[kiac035-B71] Yu XH , Chen MH, Liu CJ (2008) Nucleocytoplasmic-localized acyltransferases catalyze the malonylation of 7-*O*-glycosidic (iso)flavones in *Medicago truncatula*. Plant J 55: 382–396 18419782 10.1111/j.0960-7412.2008.03509.x

[kiac035-B72] Zhou S , Runge T, Karlen SD, Ralph J, Gonzales-Vigil E, Mansfield SD (2017) Chemical pulping advantages of Zip-lignin hybrid poplar. ChemSusChem 10: 3565–3573 28768066 10.1002/cssc.201701317 PMC5697620

[kiac035-B73] Zhu Y , Regner M, Lu F, Kim H, Mohammadi A, Pearson TJ, Ralph J (2013) Preparation of monolignol γ-acetate, γ-*p*-hydroxycinnamate, and γ-*p*-hydroxybenzoate conjugates: Selective deacylation of phenolic acetates with hydrazine acetate. RSC Adv 3: 21964–21971

