# Supplementary material for: Identification and characterization of a set of monocot BAHD monolignol transferases
Source: Plant Physiol. 2022 Feb 3;189(1):37–48. doi: 10.1093/plphys/kiac035 (PMC9070852; doi:10.1093/plphys/kiac035)
Supplement: kiac035_Supplementary_Data [file kiac035_supplementary_data.pdf]

Identification and Characterization of a Set of Monocot BAHD Monolignol Transferases

Rebecca A. Smith,[a*] Emily T. Beebe,[a,b] Craig A. Bingman,[b] Kirk Vander Meulen,[a,b] Alexis Eugene,[a] Alexander J. Steiner,[a,1] Steven D. Karlen,[a] John Ralph,[a,b] and Brian G. Fox[b]

[a]Great Lakes Bioenergy Research Center, University of Wisconsin-Madison, Madison, Wisconsin 53726 USA

[b]Department of Biochemistry, University of Wisconsin-Madison, Madison, Wisconsin 53706 USA

Corresponding author: Rebecca A. Smith, rasmith29@wisc.edu

One Sentence Summary:

A group of identified BAHD acyltransferases function as feruloyl-CoA monolignol transferases and/or *p*-coumaroyl-CoA monolignol transferases *in vitro* and *in planta*.

Funding:

This work was supported by the Department of Energy Great Lakes Bioenergy Research Center DOE BER Office of Science DE-SC0018409, and by the US Department of Energy, Office of Science, Basic Energy Sciences under award no. DE-SC0020349 to B.G.F.

Supplemental Data

Supplemental Table S1. Cloning and genotyping primers, and full coding sequences for FMT and PMT genes of interest.

**Cloning primers**	**F primer**	**R primer**
AsFMT	5'- ATGACGATCATGGAGGT -3'	5'- CTAGGAAGCGAAAGCAG -3'
OsFMT	5'- ATGGTCGCTGTCACC -3'	5'- CTATATGAAGGCCTTCATCTC -3'
SbFMT	5'- ATGGCGACGACCATC -3'	5'- CTAAAAGAAGGCCTTCATCTC -3'
PvFMT	5'- ATGGTGAACATCACCGTG -3'	5'- CTAAAAGAAGGCCTTCATCTC -3'
ZmFMT	5'- ATGGCGAGCATCACC -3'	5'- CTAAAAGAAGGCTTTCATCTC -3'
SbPMT	5'- ATGGGCACAATCGATGATA -3'	5'- CTATGAGCAGGGCTGG -3'
PvPMT	5'- ATGGGTACCATCGGGT -3'	5'- CTACTGGTGGTTCCCG -3'
Adaptor attB	5'-GGGGACAAGTTTGTACAAAAAAGCAGGCTT-3'	5'-GGGGACCACTTTGTACAAGAAAGCTGGGTG-3'
**Genotyping primers**	**F primer**	**R primer**
AsFMT	5'- TGACGATCATGGAGGTTCAA -3'	5'- CGCGAATATTGTGGATGTTG -3'
OsFMT	5'- GGATGTTGCCACCAAAGAGT -3'	5'- GTCATAATCCGGGTCCCTTT -3'
SbFMT	5'- TGCTGAGGGTGTTGCTAGTG -3'	5'- TGGTACACCCCACCCATAGT -3'
PvFMT	5'- ACGCGATTTCTGAGTTTGCT -3'	5'- CTGGAACCCAAAGGACTTGA -3'
ZmFMT	5'- GCTTGTGGAGGATTTGTGGT -3'	5'- AATGTTTGAACCCGAAGCAC -3'
SbPMT	5'- AGTGGGTTCTACGGCAACTG -3'	5'- GTCCAGTCGGAGACGAAGAG -3'
PvPMT	5'- CCAAGGTCAAGTCCAGCTTC -3'	5'- GAAGCAGTTGCCGTAGAACC -3'
Actin	5'- CCAGAAGGATGCATATGTTGGTGA -3'	5'- GAGGAGCCTCGGTAAGAAGA -3'
**Gene sequences**		

AsFMT<br>XM_017385232.1	ATGACGATCATGGAGGTTCAAGTTGTATCTAAGAAGATGGTAAAGCCATCAGTTCCGACTCCTGACCACCACAAGACTT<br>GCAAATTGACGGCATTCGATCAGATTGCTCCTCCGGATCAAGTTCCCATTATTTACTTCTACAACAGCAGCAACATCCA<br>CAATATTCGCGAGCAATTGGTAAAATCCTTGTCCGAAACTCTAACCAAGTTTTATCCATTAGCTGGAAGATTTGTTCAA<br>GATGGTTTCTATGTCGATTGTAATGATGAAGGGGTCTTGTACGTAGAAGCTGAAGTTAACATTCCGCTAAACGAATTCA<br>TCGGACAAGCAAAGAAAAATATACAACTTATCAATGATCTTGTTCCGAAAAAAAACTTCAAGGATATTCATTCATATG<br>AAAATCCAATAGTGGGATTACAGATGAGTTATTTCAAGTGTGGTGGACTTGCTATTTGCATGTATCTTTCGCATGTTGTA<br>GCTGATGGATATACAGCAGCAGCATTCACTAAAGAGTGGTCTAACACAACCAATGGCATCATCAATGGCGATCAACTA<br>GTTTCTTCTTCTCCGATTAACTTCGAATTGGCAACTCTAGTCCCAGCTAGAGATTTATCGACGGTGATCAAGCCAGCCGT<br>GATGCCACCATCAAAGATCAAGGAAACCAAGGTTGTCACAAGGAGGTTTCTGTTCGATGAAAATGCGATATCAGCTTT<br>CAAAGACCATGTCATCAAATCCGAAAGCGTTAACCGGCCTACACGGGTGGAAGTTGTGACATCTGTGTTATGGAAGGC<br>TCTGATCAACCAGTCTAAGCTTCCAAGTTCTACACTATATTTTCACCTCAACTTTAGAGGGAAAACAGGCATCAACACC<br>CCACCGCTAGATAATCATTTTTCGCTTTGCGGAAACTTTTACACTCAGGTTCCTACAAGGTTCAGGGGGGGAAATCAAA<br>CAAAACAGGATTTGGAATTGCATGAATTGGTCAAGTTGTTGAGAGGAAAGTTGCGTAACACTCTGAAGAATTGCTCCG<br>AAATTAACACTGCCGATGGGCTGTTCCTGGAAGCAGCTAGTAATTTCAATATTATACAGGAAGATTTGGAGGACGAAC<br>AAGTGGATGTTCGGATTTTTACAACGTTGTGTAGGATGCCTTTGTATGAAACTGAGTTTGGGTGGGGAAAACCAGAATG<br>GGTTACCATTCCAGAGATGCATTTGGAGATAGTGTTTCTTTTGGACACTAAATGTGGGACTGGTATTGAGGCATTAGTG<br>AGCATGGATGAAGCAGATATGCTTCAGTTTGAACTTGATCCCACCATCTCTGCTTTCGCTTCCTAG
OsFMT<br>XM_015785190.2	ATGGTCGCTGTCACCGTGATGAGGAAGTCCCGGAACTTCGTCGGGCCGTCTCCTCCGACGCCGCCGGCCGAGATCACG<br>ACGACGCTCGAGCTGTCGTCCATCGACCGCGTGCCCGGGCTGCGCCACAACGTGCGGTCCCTGCACGTGTTCCGCCGCC<br>ACAAGAACAGCGGGCCCGTCGTCGACGGTGATAGCAGGAGGCCGGCCGCCGTGATCCGCGCGGCGCTCGCCCGGGCG<br>CTGGCGGACTACCCGGCGTTCGCCGGCCGATTCGTCGGCTCCCTGCTGGCCGGCGACGCCTGCGTCGCGTGCACCGGCG<br>AGGGCGCGTGGTTCGTGGAGGCAGCCGCGGACTGCAGCCTCGACGACGTGAACGGCCTCGAGTACCCGCTCATGATCT<br>CCGAGGAGGAGCTGCTGCCTGCCCCCGAGGACGGCGTCGACCCTACCAGTATTCCAGTCATGATGCAGGTGACTGAAT<br>TCACTTGTGGAGGATTTATCTTGGGCCTTGTGGCAGTCCACACCCTTGCTGATGGACTTGGAGCAGCACAATTCATCAC<br>TGCAGTAGCTGAATTGGCCCGTGGCATGGACAAGCTCAGGGTGGCTCCCGTGTGGGATCGCTCGCTGATACCGAACCC<br>ACCTAAGCTCCCTCCTGGCCACCACCATCGTTCCAGTCCTTTGGTTTTCAGCATTTCTCCACAGATGTCACCTCTGACC<br>GTATAGCTCACGTGAAGGCTGAGTACTTCCAGACCTTTGGCCAGTATTGTTCCACCTTTGATGTTGCTACTGCTAAGGTT<br>TGGCAGGCCAGGACACGGGCCGTCGGGTACAAACCGGAGATCCAGGTCCATGTGTGTTTCTTTGCAAACACGCGTCAC<br>CTGCTCACGCAGGTTCTCCCAAAAGATGGGGGCTACTATGGCAACTGCTTTTATCCAGTGACTGTGACAGCAATAGCTG<br>AGGATGTTGCCACCAAAGAGTTGCTTGATGTGATCAAGATAATTCGGGATGGAAAGGCGAGGCTCCCCATGGAGTTTG<br>CAAAGTGGGCTTCAGGGGATGTGAAAGTTGATCCCTACGCATTGACATTTGAACACAATGTGCTTTTTGTGTCTGATTG<br>GACGAGGTTAGGATTCTTCGAGGTAGACTATGGGTGGGGTACACCTAATCACATCATACCATTCACTTATGCAGACTAC<br>ATGGCAGTCGCAGTGCTTGGTGCTCCACCAATGCCAAAGAAAGGACCCGGATTATGACACAGTGTGTGGAGAACAAG<br>TGTATCAAGGAGTTCCAAGATGAGATGAAGGCCTTCATATAG
SbFMT<br>XM_002441921.2	ATGGCGACGACCATCATCACGGTGACAAGGAAATCCCAGTCGTTCGTCGTGCCGTCGTCGTCGTCCGCGCCGGTGCCGA<br>CGACGGCCGAAACGCTGGAGCTGTCGGCCATCGACCGCGTGCCGGGGCTGCGCCACACGGTGCGGTCCCTGCACGTGT<br>TCCGCCGCAAGGCGGACGACGACGCCGCCGCCGCCGCCGCTGCTGCCAGCAGGAGGCCTGCGGAGGTGATCCGGGCA<br>GCGCTGTCCCGCGCTCTGGTGGACTACCGTCCGTTCGCCGGCCGCTTCGTCGGCTCGCTGTACGCCGGGGAGGCGTGCG<br>TCGAGTGCACCGACGAGGGCGCCTGGTTCGTGGAGGCCGTCGCTGACTGCAGCCTCGATGACGTGAACGGCCTCGACG<br>ACTACCCGCTCATGGTCTCCGAGGAAGAGCTGCTGCCGGCCCCAGAGGAAGGTGTTGACCCTACCAGTATTCCCATGAT<br>GATGCAGGTCACGGAATTTTCTTGTGGAGGATTTGTGGTGGGGCTGGTCGCAGTCCACACCCTTGCAGATGGGCTCGGT<br>GCAGCTCAATTCATCAATGCAATTTCCGAGTTTGCCCGTGGACTAGATAAACTTACAATAGCACCTGTGTGGGCTCGGT<br>CGTTAATACCAAACCCACCTAAGCTGCCTCCTGCGCCGCCACCATCCTTTGAGTCCTTTGGGTTCAAACATTTTGTCATG<br>GATGTTACTTTTGACAATATTGCACATGTCAAGACTGAGTACTTTCAAGCCAATGGACAATACTGCTCTACATTCGATG<br>TTGCCATTGCCAAGGTTTGGCAAGCTAGGACCAGGGCAATCAAGTACAATCCAGATGTCAAGGTCCATGTTTGCTTCTT<br>TGCCAACACTCGCCACCTCCTCACACGGGAGCTTCCAAACGATGGGGGCTTCTATGGAAATTGCTTCTATCCGGTGACT<br>GTAACAGCAACTGCTGAGGGTGTTGCTAGTGGAGGATTGCATGATGTGATTAGGATGATACGGGATGGGAAGGCTAGG<br>CTGCCTTTGGAGTTTGCCAAATGGTCCATGGGTGATGTGAAGGTAGACCCATATCAACTGACATTCAAGCACAATGTTC<br>TGTTTGTGTCTGATTGGACGAGGCTTGGATTCTTTGAGGTTGACTATGGGTGGGGTGTACCAAACCATATCATACCTTTC<br>ACTTATGCAGACTACATGGCTGTAGCAGTTCTTGGGGCTCCACCTACTACAGTGAAGAACAAGGGGACTCGAATAATG<br>ACACAGTGCGTGGAGGAGAAGCATCTCATGGAATTCAAGGATGAGATGAAGGCCTTCTTTTAG
PvFMT<br>Pavir.3KG495300	ATGGTGAACATCACCGTGACAAGGAAATCCCAGTCCTTCGTCGTGCCGGCGTCGTCCGAGCCGGCGTCGGCCGAGACG<br>ACGCTCGAGCTATCGGCGATCGACCGCGTGCCGGGCCTCCGCCACACGGTGCGGTCGCTGCACGTGTTCCGCAACAAG<br>AAGGAGTCCGCCGCAGGCGCCGGCTGCGACGACGACGATGCTGCCAGCAGGCCGGGGGAGGTGATCCGCGCGGCGCT<br>GTCCCGCGCGCTGGTGGATTACCGCCCGTTCGCCGGCCGCTTCGTCGGCTCGGTCGCCGCCGGGGAGACCTGCGTCGAG<br>TGCACCGACGACGGCGCGTGGTTCGTGGAGGCCGTCGCCGACTGCAGTCTCGAGGGCGTGAATGGCCTCGACTACCCG<br>CTCATGGTCTCCGAGGAAGAGCTGCTGCCCGCTCCAGAGGAAGGCGTTGACCCTACAAGTATTCCGATCATGATGCAG<br>GTTACAGAATTTGCATGCGGAGGATTTGTGGTTGGGCTGGTAGCAGTCCACACTCTTGCTGACGGGCTCGGCGCCGCCC<br>AATTCATCAACGCGATTTCTGAGTTTGCTCGTGGGATGGAAAAGCCCACGGTAGCACCCGTATGGGCTCGGGCTTTAAT<br>ACCAAACCCACCCAAACTGCTTCCCGGGGCACCACCGTCCTTCAAGTCCTTTGGGTTCCAGCACTTCACCGTGGATGTG<br>ACCTCTGACCGGATTGCCTACGTCAAGACCCAGTACCATCAGGCCACTGGACAGTACTGCTCCACCTTTGATGTCGCCA<br>TTGCCAAGGTTTGGCAGGCAAGAACCAAGGCAATCAAGTACAGCTTGGAGTCCCAAGTTCATGTCTGCTTCTTCGCCAA<br>CACCCGCCACCTCCTCACCCAGGTGCTGCCCAAGAATGGGGGATTCTATGGCAACTGCTTCTACCCAGTTTCTGTGACG<br>GCCACTGCTGAGGATGTTGTCACTGCAGGGTTGCTTGATGTGATCAGGATGATAAGGAATGGGAAGGCCAGGCTTCCC<br>CTGGAGTTTTCCAAGTGGGCAGCAGGGGATGTGAGTGTGGATCCATACCAGTTGACATTTGAGCACAACGTGTTGTTTG<br>TGTCTGATTGGACGAGACTTGGGTTCTCCGAGGTTGACTATGGGTGGGGTGCACCGGATCATATCGTGCCATTCACCTA

	TGCAGACTACATGGCGGTGGCGGTTCTTGGGGCTCCGCCTTCGCCGAAGAAGGGAACTCGGATTATGACGCAGTGTGT<br>GGAGGAGAAGCACCTCATGGACTTCAAGGATGAGATGAAGGCCTTCTTTTAG
ZmFMT<br>Zm00001d035246_T001	ATGGCGAGCATCACCGTGACAAGGAAATCCCAATCCTTCGTCGTGCCATCGTCCACGCCAACTCCGACGACCGAGACG<br>CTCGAGTTGTCGCCCATCGACCGCGTTCCAGGCCTGCGCCACACGGTGCGATCCCTGCACGTGTTCCGCCGCAAGGACG<br>CCGCCGCCTCCGCCGCCCACTACGATGCTGCTGCCGCCGGCAGGCCGGCCGAGGTGATCCGCGCGGCGCTGTCCCGCG<br>CGCTGGTGGACTACCGCCCGTTCGCCGGCCGTTTCGTCGGCTCACTGTACGCCGGGGAGGCGAGCGTTGAGTGCACCGA<br>CGACGGTGCGTGGTTCGTGGACGCTGTCACAGATTGCAGCCTCGAGGACGTGAACGGCCTCGACTACCCGCTTATGGTC<br>TCCGAGGAAGAGCTGCTGCCGGCTCCAGAGGAAGGTGTTGACCCAACCAGTATTCCGATTATGATGCAGGTCACGGAA<br>TTTGCTTGTGGAGGATTTGTGGTGGGGCTAGTCGCAGTGCACACCCTTGCTGACGGGCTCGGTGCAGCTCAATTCATCA<br>ATGCAATTTCTGAGTTTGCCCGTGGAGTAGTTAAACCTACAATAGCACCTATATGGGCACGGGAGTTAATACCAAACCC<br>ACCTAAAATGCCTCCTGGGCCACCACCATCCTTCGAGTGCTTCGGGTTCAAACATTTTGTTATGGATGTGGCAGTTAAC<br>AATATTGCACATGTCAAGAGTGAATACTTTCAAACCAATGGACACTATTGCTCTACATTTGATGTTGCCATTGCCAAGG<br>TTTGGCAAGCTAGGACAAGGGCAATCAAGTACGAACCAAATTTCAAGGTGCATGTTTGCTTCTTTGCCAACACTCGCCA<br>CCTCCTCACACATGTGCTACCCAAGGTTGGTGGCTTCTATGGAAATTGCTTCTATCCAGTGACTGTCACAGCAACTGCTG<br>AGGTAGTTGCTAGTTCAAGATTGCTTGATGTGATTAGGATGATAAGGGATGGGAAGGCTAGGCTTCCTTTAGAGTTTTC<br>CAGATGGTCCACGGGCAATGTGAAAGTAGACCCATATCAACTAACATTCAAGCACAATGTTCTATTTGTGTCCGATTGG<br>ACACGGCTTGGATTCTTTGAAGTTGACTATGGGTGGGGTGTACCAAACCATATCCTCCCTTTCACTTATGCAGACTACAT<br>GGCTGTAGCAGTTCTTGGAGCTCCACCGTCTATGAAGAAGGGGACTCGAATAATGACACAATGTGTCGAGGAGGAGCA<br>TCTCGTGGACTTCAAGGCCGAGATGAAAGCCTTCTTTTAG
BdFMT<br>XP_003575887.1	ATGGCAGAAATCTGCACCGTGAACAGGAAGTCCCAGTCCTTCGTCAAGCCGGCCGCGCCAACGCCAACGCCTCAGACG<br>CCGCCGCCGCTGCTGGAGCTGTCGGCCATCGACCGCGTGCCCGGGCTGCGCCACACCGTGCGCTCTCTCCACGTCTTCC<br>GCCCGCCGCCGCACGGCGACGGCGCCGCCTGCAGCAGGCCGGCCGAGGTGATCCGCGCCGCGCTGGCCCGCGCGCTCG<br>TGGAGTACCCCGCGTTCGCCGGGCGGCTCGTCGTCGGCGGCTCCGGCTCGGACTGCGGCGTGGCGTGCACCGGCGACG<br>GAGCGTGGTTCGTTGAAGCGGCCGCCGGCTGTAACCTGGAGGACGTGAACGAGCTGGACTACCCTCTCGTGGTCTGCG<br>AGGAGGAGCTGCTCCCCACCGCCCCTGAGGGAGAGCTGGATCCTACAAGCATTCCGGTCATGATGCAGGTGACCGAAT<br>TCAGCTGCGGAGGATTTGTGGTGGGCCTGGTAGCAGTCCACACCTTCGCAGACGGGCTCGGCGCGGCCCAATTCATCA<br>ACGCCATCGCCGAATTCGCCCGTGGCCTAAACAGGCCCACAGTGAATCCCATATGGGCCCGAGCCACAATCCCCAACC<br>CGCCCAAATTCCCTCCCGGCCCACCACCATCCTTCCAATCCTTCGGCTTCCAGCATTTCGCCACGGACATCCGTCCAGAC<br>CGCATCGCCCACGCCAAAGCCGAGTACCTCAAGGCCACGGGCACCCACTGCTCGGCCTTCGACGTCGCCGTGGCCAAG<br>GTCTGGCAGGCCCGAACCCGGGCCGTAAGGTACGGCCCAGAGGCCCAGGTGCAGGTCTGCTTCTTCGCCAACACGAGG<br>CACCTTCTCGGAGAGCTTCTCCCCGAAGGTTTCTACGGCAACTGCTTCTTCCCGGTCACCGTGAAGGCCAGAGCTGGGG<br>ATGTTGCCGGCAGCAAGGATTTACTTGGTATTATCCGGATGATCAGGGACGGGAAGGCCAGGCTGCCTTTGGAGTTCGC<br>CGATTGGGCGTCAGGTTTAGGAGGAGGAGGGGCTGGGGATAAGATGAAGTTTGTGCAGGATGATCCTTATGAGCTGAG<br>GTTTGAGCATAATGTGTTGTTTGTGTCGGATTGGACGAGGCTTGGGTTCTTGGAGGTGGACTATGGCTGGGGCGTGCCT<br>AGCCATGTTATACCTTTCAATTATGCGGACTACATGGCGGTCGCGGTGCTCGGTGCTCCGCCGGCGCCGGTGAAGGGGA<br>CTCGGGTCATGACGCAGTGCGTGGAGGAGAAGCATCTTAAGGAGTTCAGGGATGAGATGGAAGGCTCCTTTTAG
SbPMT<br>XM_002439193.2	ATGGGCACAATCGATGATACCGCCGGGTTATTCCCGGTGACGAGGACGAACAGGTCGCTGGTGCCGCCGTCGTCGGCG<br>ACGCCGCAGGAGACGCTGCGCCTGTCGGTGATCGACCGCGTGGCGGGGCTGCGCCACCTGGTGCGGTCGCTGCACGTG<br>TTCGCCGGCGGCGAGAACAAGAAGCAGGCGGCGCCGCCGGCGAAGTCCCTGCGGGAGGCGCTGGGAAAGGCGCTGGT<br>GGACTACTACCCGTTCGCGGGGCGGTTCGTGGAGGAAGACGGGGAGGTCCGGGTGGCGTGCACCGGCGAGGGCGCCT<br>GGTTCGTGGAGGCCGCCGCCGCGTGCTCCCTGGAGGAGGTCCGGCACCTGGACCACCCCATGCTCATCCCCAAGGAGG<br>AGCTGCTGCCGGAGCCGGCGCCCGGCGTCAACCCGCTCGACATGCCGCTCATGATGCAGGTGACGGAGTTCACGTGCG<br>GCGGCTTCGTGGTGGGTCTAATCTCCGTCCACACCATCGCCGACGGTCTAGGCGCCGGGCAGTTCATCAACGCGGTGGC<br>GGACTACGCCCGTGGCGGCGCCACCGCCGGCGCCGTCACCAGACCCCGCATCACCCCGATCTGGGCGCGCGACGTGAT<br>CCCGGACCCGCCCAAGATGCCGGCGCCGCCGCCGCGCCTCGACCTGCTGGACCTGGTCTACTTCACGACGGACCTGAG<br>CCCGGACCACATCGCCAAGGTCAAGTCCAGCTACCTCGAGTCCACGGGGCAGCGCTGCTCGGCGTTCGACGTGTGCGT<br>GGCGCGCACCTGGCAGGCCCGCGTCCGCGCGCTCCGCCTCCCGGACGCCGCCGCGCCCGTCCACGTCTGCTTCTTCGCC<br>AACACCCGCCACCTGCTCCCGGCGACGGCGGCGGCGCCGGCGAGTGGGTTCTACGGCAACTGCTTCTACACCGTCAAG<br>GCGACGCGGCCCAGCGGCGAGGTGGCGGCGGCCGACATCGTCGACGTCGTGCGCGCCATCCGGGACGCCAAGGCGAG<br>GCTCGCCGCCGACTTCGCGAGGTGGGCGGCGGGCGGGTTTGATCGGGACCCCTACGAGCTCACCTTCACCTACGACTCC<br>CTCTTCGTCTCCGACTGGACGAGGCTAGGGTTCCTCGAGGCTGACTATGGCTGGGGCACGCCGACGCACGTCGTGCCGT<br>TCTCGTACCACCCGTTCATGGCCGTCGCCGTCATCGGGGCGCCGCCGGCGCCTAAGCCCGGCGCACGCATCATGACCAT<br>GTGCGTCCAGGAGCAGCACCTGCCTGAGTTCCAGGAGCAGATGAACCAGCCCTGCTCATAG
PvPMT<br>XM_039943249.1	ATGGGTACCATCGGGTTCCCGGTGACGAGGACGAGCAGGTCGCTGGTGGCGCCGTCGTCGGCGACGCCGCAGGAGACG<br>CTGCACCTGTCGGTGATCGACCGCGTGGCGGGGCTGCGGCACCTGGTGCGGTCGCTGCACGTGTTCGACGGCCGCCGC<br>GGCGAGGCGGCGGTGAGGACGCCGGCGGAGACGCTGCGGGCGGCGCTGGGGAAGGCGCTGGTGGACTATTACCCGCT<br>GGCGGGGCGGTTCGTGGAGGAGGACGGGGAGGTGCGGGTGGCGTGCACGGCGGGGGGCGCCTGGTTCGTGGAGGCGG<br>CGGCGGCGTGCACCCTGGAGGAGGTGAAGCACCTGGACCACCCCATGGTCATCCCCAAGGAGGACCTGCTGCCGGAGC<br>CGGCGCCGGACGTCAACCCCCTCGACATGCCGCTCATGATGCAGGTGACGGAGTTCGCGTGCGGCGGCTTCGTGGTGG<br>GCCTCATCTCCGTGCACACCATCGCCGACGGCCTGGGCGCCGGGCAGTTCATCAACGCGGTGGCGGACTACGCGCGTG<br>GCCTCCCGAGGCCCCGCGTGCTCCCCGTCTGGGCGCGCGACGTCATCCCGGCGCCGTCCAGGATCGTGTCCCCGCCGCC<br>GCGGTTCGACCTCCTGGACCTCCGCTACTTCACCGTGGACCTCAGCCCGGAGCACATCGCCAAGGTCAAGTCCAGCTTC<br>TTCGAGGCGACGGGGCAGCGCTGCTCGGCGTTCGACGTGTGCGTCGCCAAGACCTGGCAGTCCCGCGTCCGCGCGCTC<br>CGGCTGGACGGCGACGACCCGGCGCGGCCCATCCACGTGTGCTTCTTCGCCAACACGCGGCACCTCCTGCCGCAGCTG<br>GCGCCCGGGTTCTACGGCAACTGCTTCTACACCGTGAGGGCGACGCGGCCCTGCGGCGAGGTGGCGGCGGCCGGCGTG<br>GTGGAGGTGGTGCGCGCCATCCGGGACGCCAAGGCGCGGCTGGGCGCGGACTTCGCGCGGTGGGCGGCGGGCGGGTT<br>CGAGCGCGACCCCTACGAGCTCACCTTCAGCTACGACTCGCTCTTCGTCTCCGACTGGACGCGGCTGGGGTTCCTGGAG

	GCGGACTACGGGTGGGGCGCGCCGGCGCACGTCGTGCCCTTCTCCTACCACCCCTTCATGGCCGTCGCCGTCATCGGCG CGCCGCCGGCGCCCAAGCCCGGCGCGCGTCATGACCATGTGCGTCACGGAGAAGCACCTGCCCGAGTTCCAGGAGC AGATGAACGCCTTCGCCGCCGGGAACCACCAGTAG

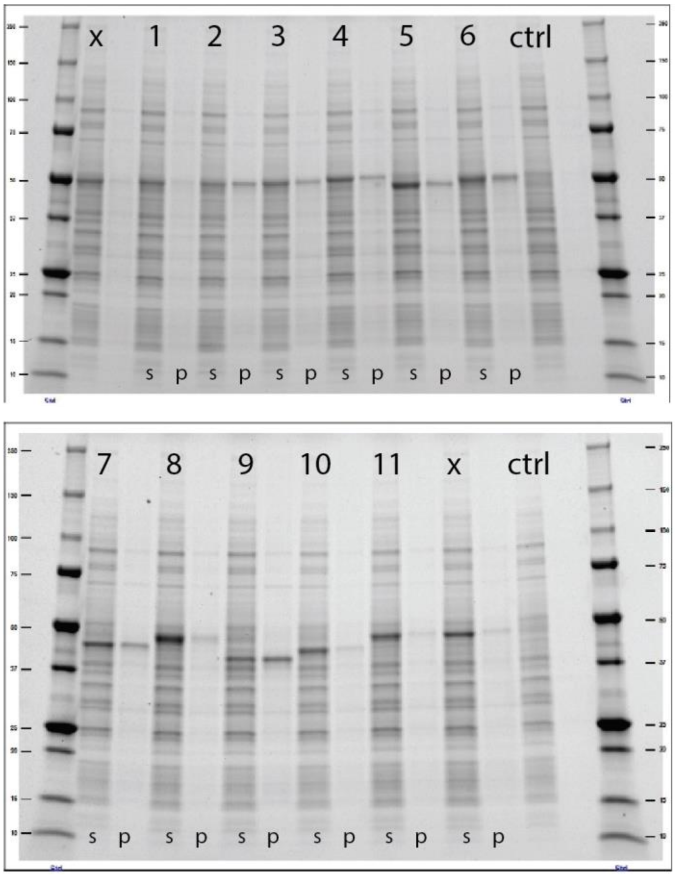

Lane	Description	Accession #	Predicted Size (Da)	# Trp	ug/rxn	µg/µL	µM	% Solubility	Projected soluble yield/mL extract
1	Angelica FMT	AHL24755	49974	4	10.7	0.43	8.6	87%	2.3
2	Corn FMT	GRMZM2G130728_P01	48020	6	13.7	0.55	11.4	45%	1.0
3	Sorghum FMT	Sb08g005680.1	48638	6	15.5	0.62	12.7	67%	1.7
4	Switchgrass FMT	Pavir.Ca02673.1	47638	6	10.8	0.43	9.1	74%	1.8
5	Rice FMT	LOC_Os05g19910.1	47652	6	14.8	0.59	12.4	74%	2.9
6	Brachypodium FMT	Bradi4g06067.1	48050	6	15.3	0.61	12.8	67%	3.1
7	Corn PMT	GRMZM2G028104_P01	47335	6	14.8	0.59	12.5	69%	2.9
8	Brachypodium PMT	Bradi2g36910.1	49434	7	15.1	0.60	12.2	87%	3.6
9	Switchgrass PMT	Pavir.J00672.1	46849	6	13.1	0.52	11.2	45%	1.3
10	Sorghum PMT	Sb09g002910.1	47500	6	9.2	0.37	7.7	92%	2.2
11	Rice PMT	LOC_Os01g18744	47170	7	10.2	0.41	8.7	93%	2.0

Supplemental Figure S1. Denaturing gels showing cell-free protein synthesis of acyltransferases and analysis of translation results. Content in gel lanes indicated in the table; “x” is not part of this study; “ctrl” is a control lane lacking acyltransferase mRNA. The # of Trp is used as a parameter in BioRad Stain-Free™ quantitative analysis. The percentage solubility is determined by comparing analysis result for soluble (s) and precipitate (p) lanes. Projected yield of soluble acyltransferase per mL of translation reaction indicated.

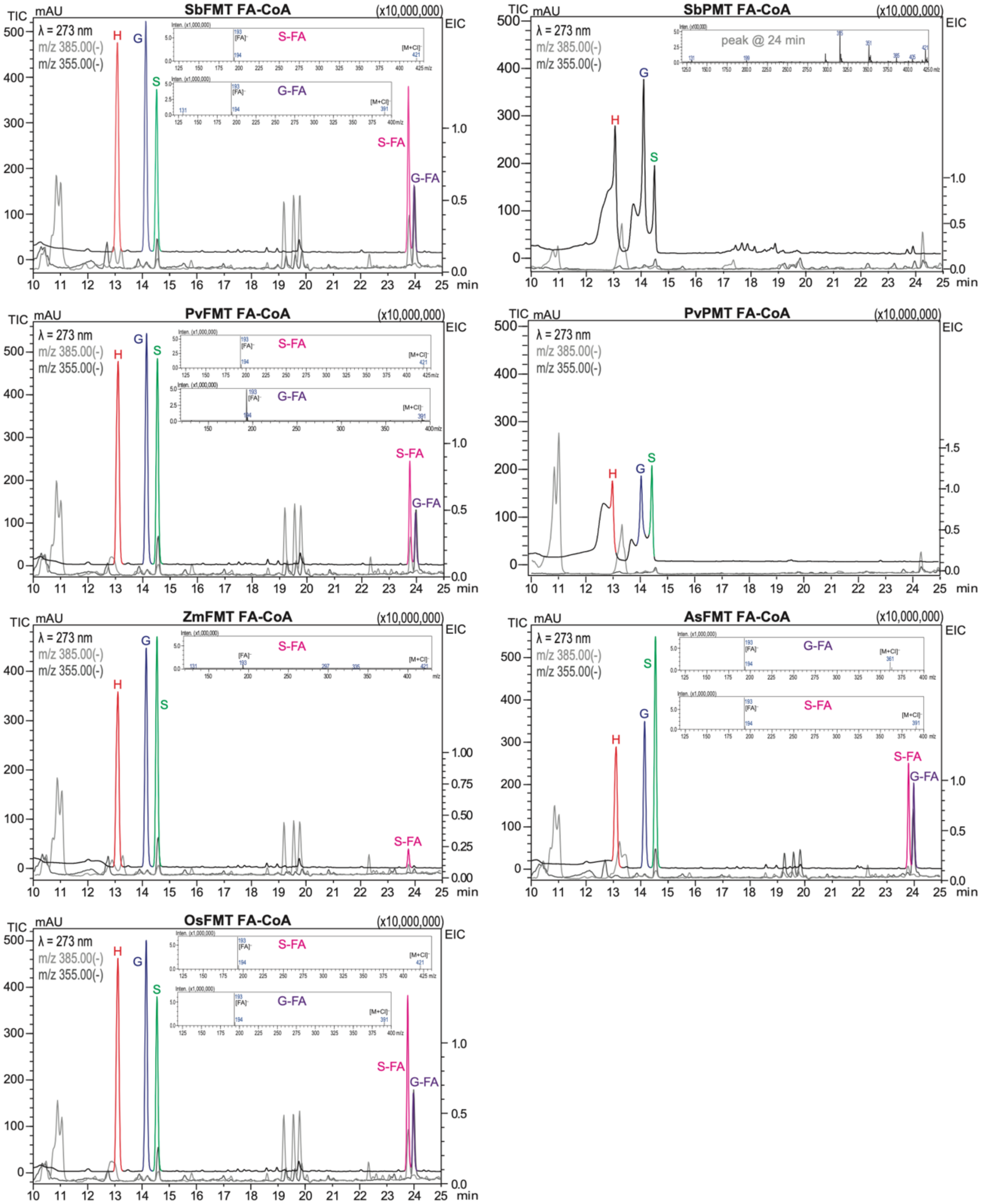

Supplemental Figure S2. Individual enzyme assays with three monolignol acceptors (*p*-coumaryl alcohol (H), coniferyl alcohol (G), and sinapyl alcohol (S)) and feruloyl-CoA as a donor. Sinapyl ferulate (S-FA; pink) and coniferyl ferulate (G-FA; purple) product formation observed in the total ion chromatograms (TIC, left axis) was confirmed by extracted ion chromatograms (EIC, right axis) at m/z 385 for S-FA and m/z 355 for G-FA. Additional support for S-FA and G-FA identities was acquired by MS product scans for each product peak, as shown in the inset of each chromatogram. Specifically, the minor peak observed at elution time of ~24 min in the SbPMT FA-CoA chromatography does not have the proper MS product scan to be a product of the acyltransferase reaction.

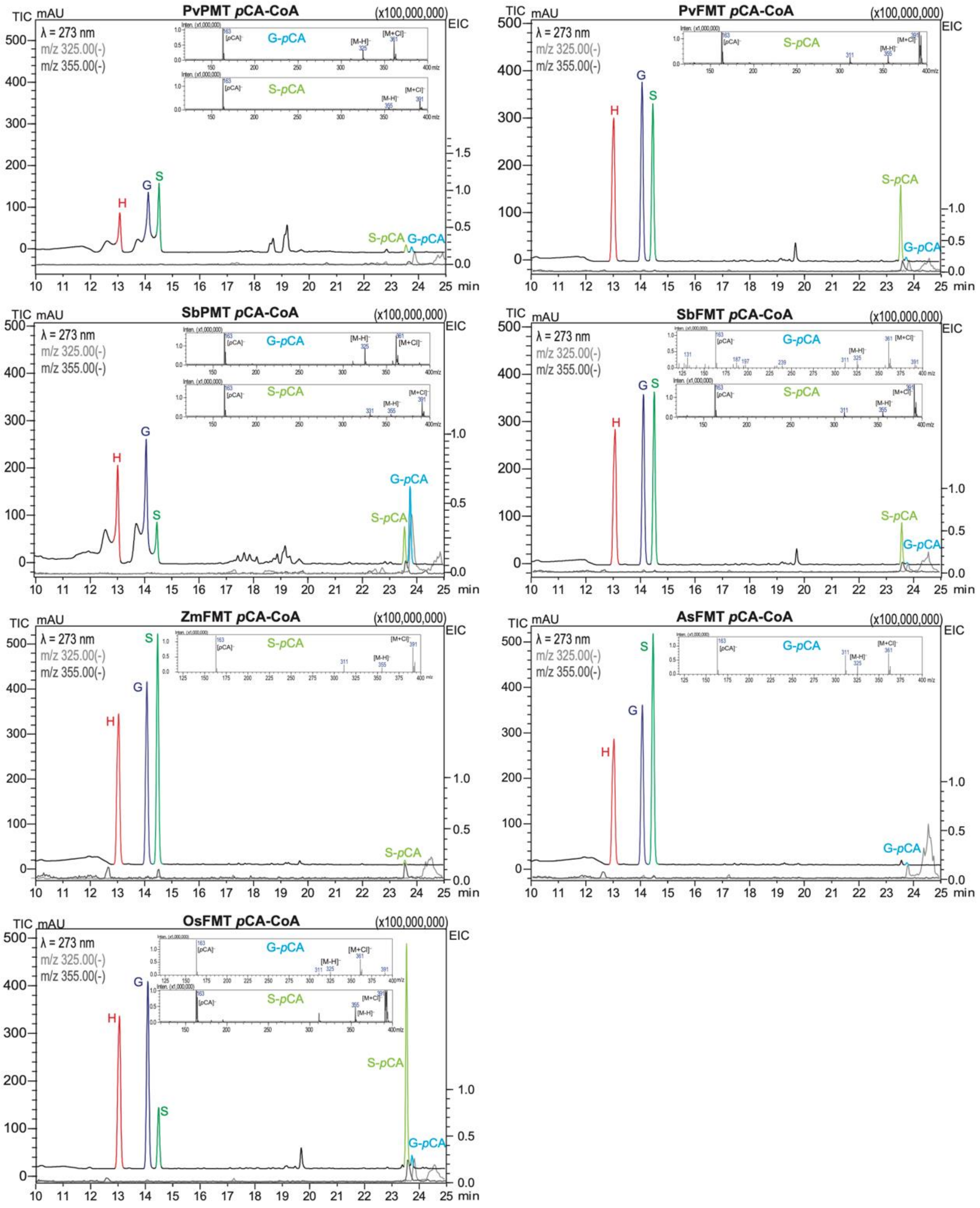

Supplemental Figure S3. Individual enzyme assays with three monolignol acceptors (*p*-coumaryl alcohol (H), coniferyl alcohol (G), and sinapyl alcohol (S)) and *p*-coumaroyl-CoA as a donor. Sinapyl *p*-coumarate (S-*p*CA; light green) and coniferyl *p*-coumarate (G-*p*CA; light blue) product formation observed in the total ion chromatograms (TIC, left axis) was confirmed by extracted ion chromatograms (EIC, right axis) at m/z 355 for S-*p*CA and m/z 325 for G-*p*CA. Additional support for S-*p*CA and G-*p*CA identities was acquired by MS product scans for each product peak, as shown in the inset of each chromatogram.

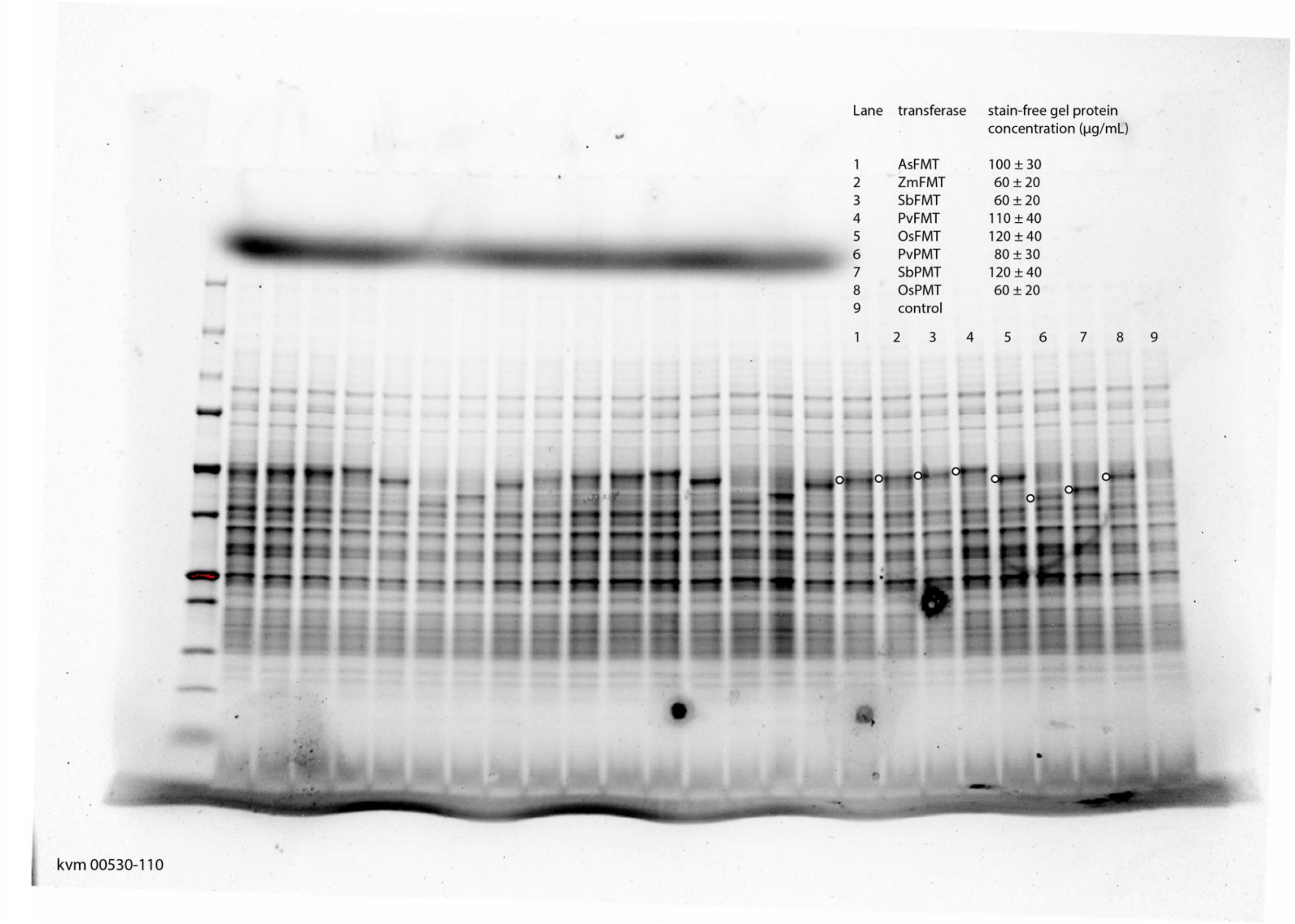

Supplemental Figure S4. BioRad Stain-Free™ denaturing gel analyses of acyltransferases translation reactions used to produce enzyme needed carry out activity measurements. Acyltransferase protein bands are marked by a white circle at the left of the gel lane. The embedded table shows the protein concentration for the marked band as determined by the stain-free analysis. Lane 9 is a control showing wheat germ extract with no added acyltransferase mRNA.

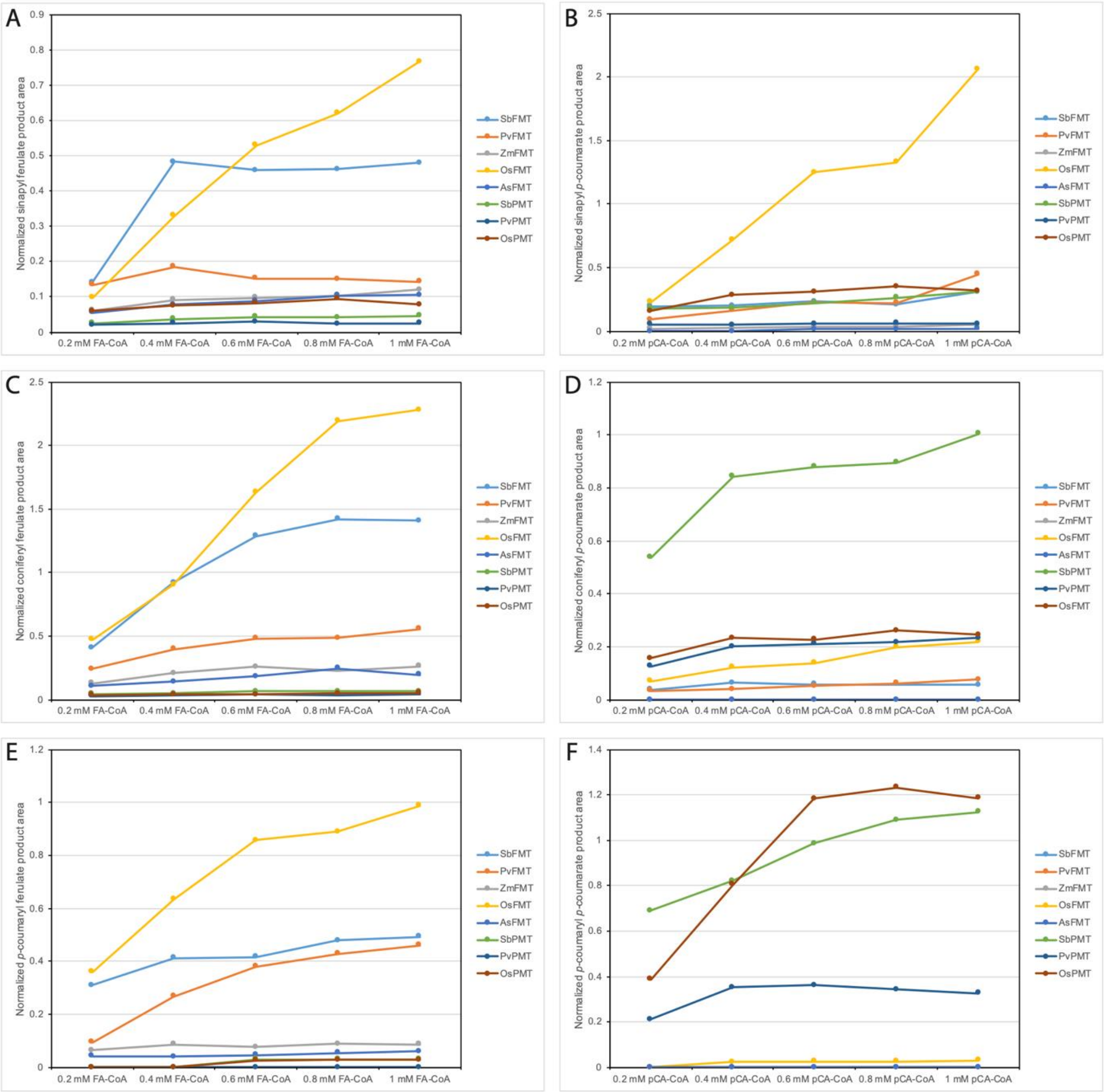

Supplemental Figure S5. Enzyme activity for the FMT and PMT enzymes. Enzymes were fed varying concentrations of CoA thioester and 1 mM sinapyl alcohol, coniferyl alcohol, or *p*-coumaryl alcohol, and the reactions were stopped after one hour. The resulting monolignol conjugate peak area was normalized to the peak area of the internal standard for sinapyl ferulate (S-FA) (A), sinapyl *p*-coumarate (S-*p*CA) (B), coniferyl ferulate (G-FA) (C), coniferyl *p*-coumrate (G-*p*CA) (D), *p*-coumaryl ferulate (H-FA) (E), and *p*-coumaryl *p*-coumarate (H-*p*CA) (F).

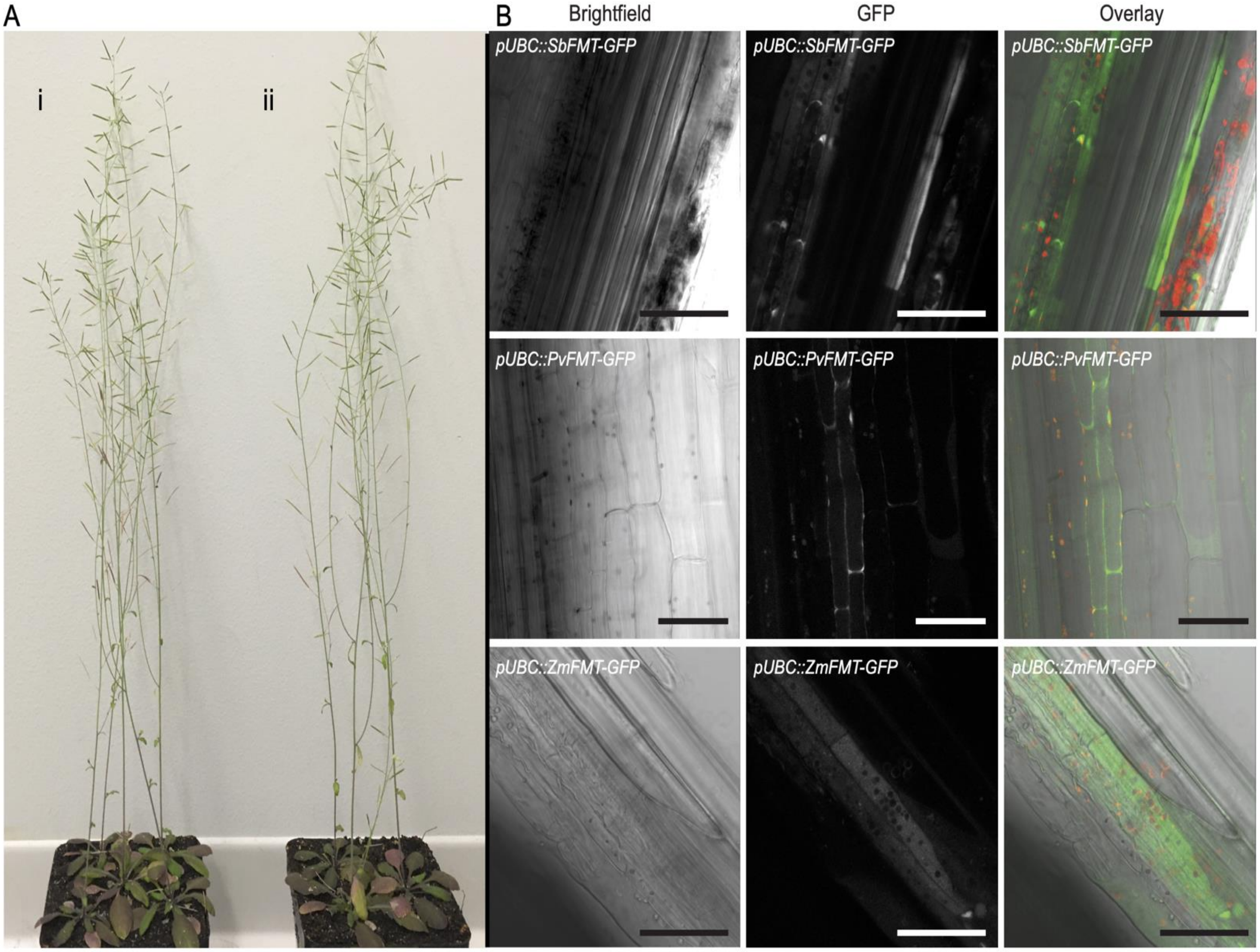

Supplemental Figure S6. Growth and localization of acyltransferase transgenic lines. (A) Examples of wild-type (i) and an FMT or PMT plant (ii) demonstrate that the presence of the transgenes does not impact normal plant growth. (B) Examples of GFP localization in transgenic lines. The FMT and PMT enzymes are localized in the cytosol. Scale bars are 50 µm.
